# Anticancer potential of *Dendrocnide meyeniana* through phytochemical profiling, ADMET analysis, molecular docking, and in silico cytotoxicity evaluation

**DOI:** 10.1038/s41598-025-32457-1

**Published:** 2025-12-27

**Authors:** Edlyn E. Pooten, Khristina G. Judan Cruz, Evaristo A. Abella, Anna Karen C. Laserna, Abul Baskhar Mir Md. Khademul Islam, Kozo Watanabe

**Affiliations:** 1 Centre for Marine Environmental Studies (CMES), Ehime University, Bunkyo-cho-3, Matsuyama, Ehime 790-8577 Japan; 2 Department of Biological Sciences, Central Luzon State University, 3120 Science City of Muñoz, Nueva Ecija, Philippines; 3 School of Arts and Sciences, Aurora State College of Technology, 3200 Baler, Aurora Philippines; 4 Central Instrumentation Facility, Office of the Vice Chancellor for Research and Innovation, De La Salle University, 2401 Taft Avenue, Malate, 1004 Manila Philippines; 5 Department of Genetic Engineering & Biotechnology, University of Dhaka, Dhaka, Bangladesh

**Keywords:** *Dendrocnide meyeniana*, GC-MS, UHPLC-QTOF-MS, In silico, ADMET, Molecular docking, Biochemistry, Cancer, Computational biology and bioinformatics, Drug discovery

## Abstract

Phytochemicals are widely explored for cancer therapeutics due to their structural diversity and broad pharmacological activities. This study investigated the phytochemical composition and anticancer potential of *Dendrocnide meyeniana* using integrated in silico approaches. Gas chromatography-mass spectrometry (GC-MS) and ultra-high-performance liquid chromatography-quadrupole time-of-flight mass spectrometry (UHPLC-QTOF-MS) identified 78 compounds, confirming the plant’s rich chemical diversity. Four cancer-related targets- EGFR, p53, MMP7 and CDK8/Cyclin C were selected for molecular docking to identify potential inhibitors. Drug-likeness and ADMET profiling of nine bioactive candidates revealed Cryptotanshinone as the most promising compound, exhibiting favorable pharmacokinetic and safety properties. Molecular docking showed that Cryptotanshinone possessed strong binding affinities toward EGFR ( -8.8 kcal/mol), p53 (-8.7 kcal/mol), MMP7 (-8.7 kcal/mol), and CDK8/Cyclin C (-9.8 kcal/mol), comparable to or exceeding the reference drug Erlotinib (-9.0 kcal/mol for EGFR). Toxicity prediction indicated no hepatotoxic, mutagenic, or cytotoxic effects, though the compound showed potential carcinogenic activity possibly linked to pathway-specific interaction in cell-cycle regulation. Molecular dynamics simulation further validated the stability of the Cryptotanshinone–EGFR complex, exhibiting moderate RMSD values and limited structural fluctuations indicative of stable interactions. Collectively, these findings highlight Cryptotanshinone from *D. meyeniana* as a promising natural lead for anticancer drug development, characterized by strong binding affinity, favorable pharmacokinetics, and structural stability in silico. Further in vitro and in vivo studies are warranted to confirm its therapeutic efficacy and safety.

**Supplementary Information:**

The online version contains supplementary material available at 10.1038/s41598-025-32457-1.

## Introduction

Plants are widely recognized as rich sources of bioactive metabolites that exhibit diverse pharmacological properties^1^. Globally, approximately 80–85% of the population relies on plant-based medicines for the management and treatment of various diseases^2,3^. Numerous plant extracts, as well as isolated bioactive compounds and their derivatives, have shown considerable potential as candidates for both the prevention and treatment of cancer^3^.

The current interest in the properties of plant compounds represents a growing area of research aimed at discovering reliable and effective anticancer agents that can selectively target tumor cells while minimizing toxicity to normal cells^4,5^. Many plant-derived compounds exert anticancer effects by modulating key cellular signaling pathways. These mechanisms include reducing oxidative stress, inducing cell cycle arrest at various checkpoints, downregulating anti-apoptotic proteins, suppressing the expression of P13K/AkT/MMP pathways, and upregulating tumor suppressor and apoptotic markers^6,7^.

*Dendrocnide meyeniana* (Walpers) Chew, *Gard. Bull. Singapore* 21: 206 (1965) is a member of the Urticaceae family and is a deciduous shrub native to the Philippines and Taiwan. It typically grows to a height of 3 to 7 m and is characterized by its large, broadly triangular leaves measuring 20 to 40 cm, densely clustered small flowers, and small fleshy fruits that range in color from white to pale violet^8^. Notably, the plant is covered with conspicuous stinging hairs, particularly on its twigs and leaf surfaces, which contain irritant compounds such as histamine, oxalic acid, and tartaric acid. These compounds can cause erythematous papules and a stinging sensation upon contact^9^. *D. meyeniana* holds cultural and medicinal importance. Traditionally, it has long been utilized by communities for its purported therapeutic properties despite its known toxicity. Phytochemical investigations have revealed the presence of diverse secondary metabolites, including saponins, phenols, tannins, flavonoids, and terpenes^10^. These bioactive constituents are known to contribute to a broad spectrum of pharmacological activities in plants, such as antibacterial, antidiabetic, antiulcer, and anticancer activities^11–16^ and anti-angiogenic effects^17^.

 In silico techniques have become essential tools in modern drug discovery and development, offering cost-effective and rapid solutions to predict pharmacokinetic properties and optimize compounds^18,19^. These computational approaches, including ADMET profiling and molecular docking, enable early assessment of drug candidates’ safety and efficacy, reducing the likelihood of late-stage failures^20^. While in vitro and in vivo methods remain crucial, in silico tools provide valuable initial insights, streamlining the drug development process and reducing reliance on expensive laboratory experiments^21^. The integration of in silico, in vitro, and in vivo techniques has shown promising results in enhancing predictive power and accelerating drug discovery^21,22^. As computational methods continue to evolve, they are revolutionizing drug design by offering rapid, cost-effective solutions that bridge the gap between discovery and development.

In this study, we identified the phytochemical constituents of *D. meyeniana* utilizing GC-MS and UHPLC-QTOF-MS analysis. We also performed predictions of their ADMET properties and molecular docking analyses to assess their binding affinity with relevant anticancer targets. Finally, we evaluated the cytotoxic potential of the identified compounds against tumor and non-tumor cell lines using in silico analyses.

## Materials and methods

### Compliance with ethical standards

This study complied with institutional, national, and international guidelines and legislation on plant research. The study adhered to the IUCN Policy Statement on Research Involving Species at Risk of Extinction (Version 1.0, approved 1989)^23^ and the Convention on International Trade in Endangered Species of Wild Fauna and Flora (CITES, 1973, with current appendices)^24^. *D. meyeniana* is currently assessed as Least Concern under the IUCN Red List Categories and Criteria (version 3.1, latest assessment 2022)^25^ and is not listed under any CITES Appendices. No endangered or protected species were involved in this research.

### Plant collection and processing

Permission to collect *D. meyeniana* was obtained from the local officials of Sta. Fe, Nueva Vizcaya, Philippines, and the Department of Environment and Natural Resources (DENR), Philippines. Voucher specimens were identified by Mr. Paul Henric Gojo Cruz, a taxonomist from the Department of Biological Sciences, Central Luzon State University, Nueva Ecija, Philippines, where the specimens are deposited in the department’s public collection with Voucher ID No. EP0001.

Matured leaves were collected from healthy plants during the early morning hours, when the photosynthetic activity is typically at its peak^18–20^. Immediately after collection, the samples were placed in a sterile polyethylene bag to prevent contamination and moisture loss. The collected material was promptly transported to the laboratory for further processing and analysis. The leaves were thoroughly washed with distilled water, followed by a final rinse with 70% (v/v) ethanol to remove surface contaminants^26^. The cleaned leaves were air-dried at room temperature for 15 days. Once completely dried, the leaves were ground into fine powder using a mechanical blender. The powdered plant materials were stored in a sterile, airtight 1000 ml container for further use.

### Ethanol extraction

Fifty (50) grams of ground leaves of *D. meyeniana* were transferred to a separate container, and 500 mL of 95% ethanol was added. The solution was kept in the dark for 72 h (3 days) at room temperature^27^. After this period, the ethanol extracted solution was separated by filtration through filter paper. Subsequently, the resulting filtrates were concentrated under reduced pressure at 40 °C using a rotary evaporator (IKA^®^ RV 10/ HB 10/ 220 to 240/ 20 to 280 rpm, China). The crude extracts were sterilized by centrifugation at 10,000 x g for 30 min, followed by membrane filtration using an acrodisc 25 mm Syringe Filter and stored at 4 °C until further use^28^.

### Gas chromatography-mass spectroscopy (GC-MS) analysis

After concentrating the extracts using a rotary evaporator, 20 µL aliquots were transferred into individual vials. The extracts were dried using a vacuum centrifuge at 25 °C for 1.5 h. Each vial was derivatized with 20 µL of methoxamine HCl in pyridine (30 mg/mL) at 37 °C for 2 h with intermittent shaking, followed by the addition of 20 µL of N, O-Bis(trimethylsilyl)trifluoroacetamide (BSTFA) with 1% trimethylchlorosilane (TMCS) and further incubation at 37 °C for 1 h. The sample solutions were then left to stand at room temperature for 1 h before GC-MS analysis. A blank was prepared following the previous procedures.

Compound identification was performed using an Agilent 8890 GC system (Agilent Technologies, Santa Clara, CA, USA) equipped with a 5977B Mass Selective Detector (MSD) and an HP-5MS column (30 m x 0.250 mm, 0.25-micron film thickness). The GC-MS operated in electron ionization mode (70 eV) with a scan range of energy method 50 to 550 m/z and a scan time of 0.2 s. A 1 µL sample solution was injected using a 1:10 ratio at an injector temperature of 300 °C. The oven was programmed to rise from 45 °C (0 min hold) to 300 °C at 10 °C/min, with a final hold for 9 min, totaling 34.5 min of routine. Peaks appearing at ~ 5.00 min corresponded to the derivatization agent. Compound identification was performed by comparing retention times, peak data, and mass spectra of detected analytes with reference entries in the NIST MS Search 2.3 library. Identification confidence was evaluated using the Match Factor (MF) and Reverse Match Factor (R. Match) criteria. The Match Factor was categorized as follows: Excellent Match (> 900), Good Match (800–900), Fair Match (700–800), and Poor Match (< 600). The Reverse Match Factor provides a complementary measure that excludes peaks present in the sample spectrum but absent in the library reference spectrum, thereby reducing spectral noise and improving identification accuracy^29^.

### Ultra-high performance liquid chromatography-quadrupole time-of-flight-mass spectrometry (UHPLC-QTOF-MS) analysis

Metabolites were also identified using UHPLC-QTOF-MS (Agilent 1260 Infinity II HPLC with 6530 QTOF-MS/MS). Samples were analyzed in negative ionization mode over a 25–1000 m/z scan range at 5 s/ spectrum and 200 ms/spectrum. A Poroshell 120 EC-C18 column (4.6 × 150 mm, 4 μm) was used with a mobile phase of ultrapure water (H2O, with 0.1% MS-grade formic acid) and acetonitrile (CAN), both containing 0.1% MS-grade formic acid^30^. For sample preparation, 250µL of H_2_O and 250µL MeOH were mixed, vortexed (1 min), sonicated (5 min), and centrifuged (12000 rpm, 5 min). A 50µL aliquot was reconstituted with the same solvent mix and processed similarly. The final 2 mL extract was transferred to vials for UHPLC-QTOF analysis. Gradient elution was carried out at a flow rate of 0.4 mL/min with a 10µL injection volume at 40 °C. Data were processed using the Agilent Mass Hunter Qualitative Analysis Navigator software (version B.08.00). Compound identification and molecular relationship analysis were performed through the Global Natural Products Social Molecular Networking (GNPS) platform. The spectral data were interpreted based on precursor and fragment ion tolerances and evaluated using cosine similarity scoring to assess spectral alignment and compound similarity^31^.

### ADMET prediction

A total of eight (8) volatile and twenty-eight (28) non-volatile compounds were selected for further analysis. Volatile compounds were chosen based on their higher relative percentage composition, whereas non-volatile compounds were selected according to their cosine similarity score and mass-to-charge (m/z) ratio, ensuring the inclusion of representative and structurally significant constituents^2,32^. The interaction of bioactive compounds with the human body involves their absorption, distribution, metabolism, excretion, and toxicity (ADMET). These properties are crucial for evaluating pharmacodynamic properties and therapeutic potential^33^. The molecular structures and SMILES notation of identified compounds were retrieved from PubChem (https://publchem.ncbi.gov, accessed May 2025), and computational approaches were done in a web-based system. Drug-likeness and pharmacokinetic properties were predicted using the SwissADME software (http://www.swissadme.ch). The toxicological profile, including oral, hepatotoxicity, cytotoxicity, mutagenicity, carcinogenicity, and immunotoxicity, was assessed via the ProTox-II tool (https://tox.charite.de/protox3 accessed May 2025)^32,34,35^.

### Molecular docking analysis

#### Ligand preparation

The bioactive constituents of *D. meyeniana* were identified as volatile and non-volatile metabolites characterized through GC-MS and UHPLC-QTOF-MS analyses (Supplementary Tables 1 and 2). The compounds selected for molecular docking were screened based on two major criteria: (i) compliance with established drug-likeness parameters, including the Lipinski rule of five and the filters proposed by Veber, Ghose, Egan, and Muegge^2^, and (ii) binding affinities below − 6.0 kcal/mol.

A total of nine (9) compounds fulfilled these criteria for molecular docking analysis. The 2D chemical structures of these selected compounds were retrieved from the PubChem database (https://publchem.ncbi.nlm.nih.gov, accessed April 2025) in Structure Data File (SDF) format. The selected compounds included: (A) Usnic acid (CID: 5646); (B) Anisomycin (CID: 253602 ); (C) 5,6,2’-trimethoxyflavone (CID: 14484690); (D) Cinchonine ( CID: 90454); (E) 8,8-dimethyl-2,10-dioxo-9 H-pyrano[2,3-f]chromen-9-yl)(Z)-2-methylbut-2-enoate (CID: 51136479); (F) 7-[(2E,5E)-7-hydroxy-3,7-dimethylocta-2,5-dienoxy]chromen-2-one ( CID:25763650); (G) 4,5-dihydroxy-4,5,6-trimethyl-2,8-dioxa-13-azatricyclo [8.5.1.013,16] hexadec-10-ene-3,7-dione (CID:56776345); (H) Cryptotanshinone (CID: 160254); (I) 6-(1,1-dimethylallyl)-2-(1-hydroxy-1-methylethyl)-2,3-dihydro-7 H-furo[3,2-G]chromen-7-one ( CID: 5287846).

The retrieved 2D structures were converted into three-dimensional (3D) conformations using Open Babel software 2.4.1 (accessed August 2025)^36^.The standard inhibitors used as positive controls were Erlotinib, a known epidermal growth factor receptor (EGFR) inhibitor^37^ and Eprenetapopt (APR-246 ), a first-in-class small molecule that reactivates p53 induces apoptosis^38^. The structures of both reference compounds were also obtained from PubChem in SDF format and subjected to the same optimization protocol. All ligand and control structures were then converted to Protein Data Bank (PDB) format using PyMOL (version 2.0) to ensure compatibility with the docking software.

Ligand optimization was performed using PyRx, which integrates AutoDock and Open Babel tools. Within Autodock, the ligand preparation settings were configured to perform necessary structural repairs and define rotatable bonds. Energy minimization was conducted using the MMFF94 force field, and the steepest descent algorithm was applied to obtain the most stable conformers. Geometry optimization and energy reduction were subsequently carried out using the same force field. The optimized structures were saved in PDB format, and polar hydrogen atoms were added while torsional bonds were assigned using Autodock tools (MGLTool 1.5.7)^39^ to prepare the ligands for molecular docking analysis.

#### Retrieval of the target protein

Target proteins were selected for molecular docking analysis based on their well-established roles in cancer progression, metastasis, and transcriptional regulation. These selected targets included the epidermal growth factor receptor (EGFR), the tumor suppressor protein p53, matrix metalloproteinase (MMP-7), and the cyclin-dependent kinase 8E/Cyclin C complex (CDK8/Cyclin C). The EGFR, particularly the tyrosine kinase domain of exon 19 deletions, is critically involved in the development and progression of non-small cell lung cancer, serving as a validated target for tyrosine kinase inhibitors. The p53 transcription factor regulates cell cycle progression, apoptosis, and genomic integrity; mutation in the TP53 gene impairs its tumor suppressor function, making it an attractive target for anticancer drug development. ^40^. Similarly, MMP7, a member of the matrix metalloproteinase family, is implicated in tumor invasion and metastasis through the extracellular matrix^41^. CDK8, in complex with Cyclin C, functions as a transcriptional co-regulator involved in oncogenic signalling pathways, including Wnt/β-catenin and p53, and its dysregulation contributes to various malignancies^42^.

The three-dimensional crystal structure of these proteins was retrieved from the Research Collaboratory of Structural Bioinformatics Protein Data Bank (RCSB PDB) (https://www.rcsb.org/, accessed 10 October 2025). The corresponding PDB identifiers and crystallographic properties were as follows: EGFR (PD ID: 4LQM) was resolved at 2.50Å (R-factors: 0.236 [Free], 0.184 [Work] and 0.190 [Observed]; p53 (PD ID: 3HF1 and 4MZ1) resolved at 1.25 Å (R-factors: 0.201 [Free], 0.198 [Work], and 0.198 [Observed]; MMP7 (PD ID: 2y6d), resolved at 1.60Å (R-factors: 0.216 [Free], 0.196 [Work], and 0.197 [Observed]; and CDK8/ Cyclin C (PD ID: 6t41) resolve at 2.45 Å (R-factors: 0.256 [Free], 0.183 [Work], and 0.185 [Observed]. All structures were derived from *Homo sapiens* and represent experimentally resolved models with high crystallographic resolution data and acceptable refinement statistics.

#### Preparation of proteins

The three-dimensional structures of the target proteins were retrieved and visualized using PyMOLv3.1^39^. Proteins were selected based on their structural completeness and biological relevance to cancer-related signaling pathways^37^. Structural optimization was performed using Biovia Discovery Studio- 2025 Client^43^. All heteroatoms, including water molecules, metal ions, and co-crystallized ligands, were removed. Hydrogen atoms were then added, and protonation states were adjusted to pH 7.0 using PROPKA^44–46^. Partial atomic charges were assigned to ensure an accurate electrostatic representation^36^. The optimized protein structures were saved in PDBQT format for subsequent molecular docking analysis.

To assess structural quality, Ramachandran plots were generated using the PROCHECK server integrated within the PDBsum database (https://www/ebi.ac.uk/thornton-srv/databases/pdbsum, accessed on 12 October 2025)^37^. The plots provide a visual evaluation of the φ (phi) and ψ (psi) torsion angles of amino acid residues, highlighting the regions corresponding to favored, allowed, and disallowed conformations.

#### Docking protocol

Molecular docking is a fundamental computational approach in structure-based drug discovery that predicts ligand binding conformations and affinities within the active sites of target proteins^47^. To evaluate the interaction potential of the selected compounds with cancer target proteins, virtual screening and site-specific molecular docking were performed. Ligand structures were first energy-minimized using the MMFF94 force field to obtain the most stable conformers^48^. The grid box parameters for docking were defined based on the amino acid residues constituting the experimentally validated binding sites of each target protein. The active sites and grid box coordinates were assigned as follows: EGFR (PDB ID: 4LQM; center X: − 59.05, Y: − 8.27, Z: − 24.77; dimensions X: 48.46 Å, Y: 65.48 Å, Z: 53.88 Å), p53 (PDB ID: 3HF1; center X: − 12.26, Y: − 37.69, Z: 3.10; dimensions X: 42.95 Å, Y: 50.94 Å, Z: 67.71 Å), MMP7 (PDB ID: 2Y6D; center X: − 25.45, Y: 8.98, Z: 9.06; dimensions X: 54.23 Å, Y: 37.77 Å, Z: 51.27 Å), and CDK8/Cyclin C (PDB ID: 6T41; center X: 4.04, Y: − 10.23, Z: 15.20; dimensions X: 81.89 Å, Y: 58.78 Å, Z: 52.56 Å). These configurations ensured that docking simulations were restricted to the biologically relevant ATP-binding pockets.

Site-specific docking was performed using PyRx v.0.8, which integrates the Autodock Vina engine for efficient ligand-protein interaction analysis^49^. Ligand torsional flexibility was maintained during docking to account for conformational variation. The resulting complexes were visualized and analyzed using PyMol (v2.0 and Biovia Discovery Studio 25 Client to examine binding poses, hydrogen bonding, hydrophobic interaction, and overall interaction profiles^32,49^.

To ensure the reliability of the molecular docking protocol, both re-docking and structural alignment approaches were employed^50^. The co-crystallized ligand of each cancer target protein complex was extracted and re-docked into its corresponding active site using identical docking parameters. The root-mean-square deviation (RMSD) between the redocked and crystallographic ligand poses was computed using RDKit An RMSD value of ≤ 2.0 Å was considered acceptable, confirming the accuracy and reproducibility of the docking parameters in reproducing experimentally observed binding orientation.

#### Protein-ligand interaction analysis

Protein-ligand interactions play crucial roles in signal transduction, immune responses, and gene regulation, thereby serving as the foundation for understanding biological regulation and identifying therapeutic targets^51^. The binding characteristics between the selected cancer-target proteins and screened ligands were analyzed using Biovia Discovery Studio v32 ^43^. Interaction analyses focused on identifying key non-covalent interactions, including hydrogen bonds, hydrophobic (π–π-alkyl and π–π) interactions, and electrostatic forces^52^. Key interaction parameters such as binding affinities, interacting residues, hydrogen bond distances, and bond angles (D-H- A) were evaluated. Particular emphasis was placed on hydrogen bond geometry, as shorter bond distances and optimal angles are indicative of stronger and more stable protein-ligand complexes.

### Molecular dynamics

The ligand exhibiting the highest affinity was subjected to molecular dynamics (MD) simulation using the Gromacs 2020.4 package. The docked complex of cancer-related protein – EGFR with Cryptotanshinone was simulated to investigate the dynamic stability, conformational flexibility, and binding persistence within the active site. The simulation was performed in triplicate for 50 ns each on the high-performance computing cluster at the Bioinformatics Resources and Applications Facility (BRAF), C-DAC, Pune with a preinstalled Gromacs 2020. The all-atom CHARMM36 force field was used to parameterize the protein, while ligand topology was generated using the Swiss Param server^37^ The complex was solvated in the dodecahedral box containing TIP3P water molecules^53^ and neutralized with appropriate Na^+^ or Cl^−^ counterions to maintain an ionic strength of 0.1 M.

Energy minimization was performed using the steepest descent and conjugate gradient algorithms for up to 50,000 steps, until the maximum force (F-max) was below 1000 kl/mol/nm, thereby eliminating steric dashes and unfavorable contacts. System equilibration was subsequently carried out in two stages: (i)NVT ensemble (constant number of particles, volume, and temperature) for temperature stabilization at 300 K using the velocity-rescaling thermostat, and (ii)NPT ensemble (constant number of particles, pressure, and temperature) for pressure stabilization at 1 bar using the Parrinello–Rahman barostat.

The conditions ensured equilibrium of system density (1023 kg/m^3^), approximating physiological conditions. The Verlet leapfrog integrator was applied for time integration^54^ while the particle–Mesh Ewald (PME) method was employed to accurately compute long-range electrostatic interactions^55^. The production MD simulation was run for 50ns with coordinates recorded every 10ps.

Post simulation, the binding free energies (ΔG_bind) of the receptor-ligand complex was estimated using the Molecular Mechanism Poisson-Boltzmann Surface Area (MM/PBSA) approach implemented in the gmx-MMPBSA tool, based on extracted trajectory frames. The binding free energy was computed as:

$$\Delta {{\mathrm{G}}_{\_{\mathrm{bind}}}}=\Delta {{\mathrm{G}}_{{\mathrm{RL}}}}--(\Delta {{\mathrm{G}}_{\mathrm{R}}}+\Delta {{\mathrm{G}}_{\mathrm{L}}})$$

where ΔG_RL, ΔG_R, and ΔG_L represent the free energies of the complex, receptor, and ligand, respectively^36^.

Furthermore, trajectory-based analyses were carried out to evaluate the stability and flexibility of the system. The root-mean-square fluctuation (RMSF) quantified residue-level flexibility. The radius of gyration (Rg) was assessed to determine protein compactness during simulation, and the number of hydrogen bonds (H-bonds) between the receptor and ligand was monitored to evaluate interaction persistence and binding strength. These analyses provide detailed insight into the dynamic behavior and conformational stability of the complex under physical conditions.

### In silico cytotoxicity prediction

The cytotoxic potential of the major compounds of *D. meyeniana* was subsequently evaluated using the in-silico cell line cytotoxicity predictor (CLC-Pred) available at http://www.way2drug.com/Cell-line/ accessed 10, October 2025. This web-based tool predicts the cytotoxic effects of chemical compounds on both human tumor and non-tumor cell lines by analyzing the structure-activity relationship. The results include two probability scores: Pa (probability of activity) and Pi (probability of inactivity). A compound is considered to have potential cytotoxic activity if Pa > Pi, indicating a higher likelihood of being active against the tested cell lines^33^.

## Results

### Phytochemical composition of *D. meyeniana* detected by GC-MS and UHPLC-QTOF-MS

GC–MS analysis of the ethanolic extract of *D. meyeniana* revealed 39 volatile compounds (Supplementary Table 1), with terpenoids, fatty acids, and other organic compounds as the major classes. Eight of these compounds, listed in Table 1, were selected based on their higher relative percentage composition for ADMET analysis. Among these, Stigmast-5-ene,3β-(trimethylsiloxy)-,24s emerged as the most abundant constituent, representing 26.99% of the total area. The second most prevalent compound was Phytol (11.735), a diterpene alcohol. Several fatty acids were also prominent, including palmitic acid (10.28%), ά-Linolenic acid (3.81%), and 9,12-octadecadienoic (linoleic acid) (4.87%). Furthermore, notable terpenoids such as Silphinene (3.75%) and Neophytadiene (2.96%) were detected.

**Table 1:** *D. meyeniana* compounds identified through GC-MS analysis used for ADMET prediction.

Peak no.	RT (min)	MW (g/mol)	Tentative compound	% Peak area	MF	Nature of compound
1	30.350	484.87	Stigmast-5-ene,3β-(trimethylsiloxy)-,24s	26.99	C_32_H_56_O	Terpenoid
2	20.068	296.50	Phytol	11.73	C_20_H_40_O	Terpenoid
3	18.855	256.42	Palmitic acid	10.28	C_16_H_36_O_2_	Fatty acid
4	20.382	280.45	9,12- octadecadienoic acid	4.87	C_18_H_32_O_2_	Fatty acid
5	20.455	278.40	ά-linolenic acid	3.81	C_18_H_32_O_2_	Fatty acid
6	11.176	204.35	Silphinene	3.75	C_1_H_245_	Terpenoid
7	18.054	291.435	2,4,6 tri-tert-butylnitrobenzene	3.74	C_18_H_36_O_2_	Organic compound
8	16.812	278.5	Neophytadiene	2.96	C_20_H_38_	Terpenoid

UHPLC–QTOF–MS profiling of *D. meyeniana* identified 39 non-volatile compounds (Supplementary Table 2), of which 28 were selected based on cosine similarity scores and mass-to-charge (m/z) ratios for ADMET prediction as listed in Table 2. The analysis showed that a chemical profiling of extracts revealed a diverse array of compounds, including alkaloids, fatty acids, flavonoids, terpenoids, and coumarins, which are known for their pharmacological significance. Among the alkaloids identified were Anisomycin, Cinchonine, and Indole-3-carboxaldehyde. Several structurally complex alkaloid derivatives were also detected, such as 3-methyl-8,10,20,22-tetraoxa-3-azapentacyclo[15.7.0.05,13.07,11.019,23]tetracosa-1(17),5,7(11),12,18,23-hexaen-14-one, and monocrotaline. The fatty acid fraction included Docosanol, 6-hydroxypalmitic acid, and 13 S-hydroxy-6Z,9Z,11E-octadecatrienoic acid (coriolic acid). Other notable fatty acids, such as 9,10-epoxy-12Z-octadecenoic acid|(+-)9(10)-, Decylbenzenesulfonic acid, 9-hydroxy-10,12-octadecadienoic acid, were also present, further contributing to the extract’s therapeutic potential. The flavonoid content, characterized by polyphenolic aromatic compounds, included 5,6,2’-trimethoxyflavone and Methyl 2-[(3,4-diethoxyphenyl)methylene]-3-oxobenzo[b]furan-5-carboxylate. Terpenoids were well represented with complex structures such as Uracaric acid and Cryptotanshinone, and highly oxygenated derivatives like 1R,2R,4aS,6aS,6bR,10 S,12aR,14bS)-1,8,10-trihydroxy-1,2,6a,6b,9,9,12a-heptamethyl-2,3,4,5,6,6a,7,8,8a,10,11,12,13,14b-tetradecahydropicene-4a-carboxylic and others. Additionally, coumarins class was marked by bioactive molecules including 8-8-dimethyl-2,10-dioxo-9 H-pyrano[2,3-f]chromen-9-yl) (Z)-2-methylbut-2-enoate and 6-(1,1-dimethyllyl)-21- hydroxy-1-methylethyl)-2,3-dihydro-7 H-furo[3,2G]chromen-7-one.

**Table 2:** *D. meyeniana* compounds based on UHPLC-QTOF-MS used in ADMET prediction.

ID	RT (min)	m/z	Tentative compound	MW (g/mol)	MF	Cosine similarity	Nature of compound
1	23.29	325.30	Docosanol	326.6	C_22_H_46_O	0.96	Fatty acid
2	19.61	343.08	Usnic acid	344.3	C_18_H_16_O_7_	0.96	Polyketides
3	21.65	297.14	Decylbenzenesulfonic acid	298.4	C_16_H_26_O_3_S	0.96	Polyketides
4	16.61	264.10	Anisomycin	265.3	C_14_H_19_NO_4_	0.96	Alkaloid
5	19.95	311.10	5,6,2’-trimethoxyflavone	312.3	C_18_H_16_O_5_	0.95	Flavonoid
6	20.24	293.16	Cinchonine	294.4	C_19_H_22_N_2_O	0.94	Alkaloid
7	23.95	271.23	16-hydroxypalmitic acid	272.42	C_16_H_32_O_3_	0.94	Fatty acid
8	20.25	293.20	13 S-hydroxy-6Z,9Z,11E-octadecatrienoic acid13(S)	294.4	C_18_H_30_O_3_	0.93	Fatty acid
9	27.35	341.10	8,8-dimethyl-2,10-dioxo-9 h-pyrano [2,3-f] chromen-9-yl) (Z)-2-methylbut-2-enoate	342.3	C_19_H_18_O_6_	0.93	Coumarin
10	23.05	483.30	1-hexadecanoyl-2-(9z-octadecenoyl)-sn-glycero-3-phospho-(1’-rac-glycerol)	749	C_40_H_77_O_10_P	0.92	Fatty acid
11	21.13	311.20	Benzenesulfonic acid, 4-undecyl-	312.5	C_17_H_28_O_3_S	0.92	Organic acid
12	10.14	144.00	Indole-3- carboxaldehyde	145.2	C_9_H_7_NO	0.89	Alkaloid
13	14.88	487.343	(1R,2R,4aS,6aS,6bR,10 S,12aR,14bS)-1,8,10-trihydroxy-1,2,6a,6b,9,9,12a-heptamethyl-2,3,4,5,6,6a,7,8,8a,10,11,12,13,14b-tetradecahydropicene-4a-carboxylic acid	488.7	C_30_H_48_O_5_	0.85	Terpenoid
14	21.65	295.20	9,10-eode|9,10-epoxy-12z-octadecenoic acid| (+-)9(10)-	296.4	C_18_H_32_O_3_	0.83	Fatty acid
15	14.80	487.30	(1R,2R,4aS,6aS,6bR,9R,10R,11R,12aR)-1,10,11-trihydroxy-9-(hydroxymethyl)-1,2,6a,6b,9,12a-hexamethyl-2,3,4,5,6,6a,7,8,8a,10,11,12,13,14b-tetradecahydropicene-4a-carboxylic acid. (Uncaric acid)	504.7	C_30_H_48_O_6_	0.83	Terpenoid
16	21.65	297.14	Decylbenzenesulfonic acid	298.4	C_16_H_26_O_3_S	0.81	Fatty acid
17	21.88	295.20	9-hydroxy-10,12-octadecadienoic acid	296.4	C_18_H_32_O_3_	0.81	Fatty acid
18	8.29	352.1	3-methyl-8,10,20,22-tetraoxa-3-azapentacyclo [15.7.0.05,13.07,11.019,23] tetracosa-1(17),5,7(11),12,18,23-hexaen-14-one	No available data	C_20_H_19_NO_5_	0.81	Alkaloids
19	17.57	535.20	(1R,2R,4 S,7 S,8R,9R,10 S,11R,12 S,13 S,14R,17R,18R,19R)-8-Acetoxy-10,19-dihydroxy-1,9,18-trimethyl-15-oxo-16,20-dioxahexacyclo [15.3.2.0 ~ 2,13 ~ 0.0 ~ 4,12 ~ 0.0 ~ 7,11 ~ 0.0 ~ 14,19~] docos-5-ene-5-carboxylic acid	490.5	C_26_H_34_O_9_	0.80	Terpenoid
20	20.67	751.30	Glc-glc-octadecatrienoyl-sn-glycerol	907.4	C_59_H_102_O_6_	0.80	Organic compound
21	20.6	559.3	Glc-octadecatrienoyl-sn-glycerol	907.4	C_59_H_102_O_6_	0.80	Organic compound
22	18.2	311.1	5,6,2’-trimethoxyflavone	312.3	C_18_H_16_O_5_	0.79	Flavonoid
23	21.65	368.10	methyl 2-[(3,4-diethoxyphenyl) methylene]-3-oxobenzo[b]furan-5-carboxylate	368.4	C_21_H_20_O_6_	0.78	Flavonoid
24	19.61	138.01	4-nitrophenol	139.11	C_6_H_5_NO_3_	0.76	Phenolic
25	30.68	324.10	4,5-dihydroxy-4,5,6-trimethyl-2,8-dioxa-13-azatricyclo [8.5.1.013,16] hexadec-10-ene-3,7-dione	314.4	C_19_H_22_O_4_	0.77	Alkaloid
26	29.03	134.90	7-[(2E,5E)-7-hydroxy-3,7-dimethylocta-2,5-dienoxy]chromen-2-one	314.4	C_19_H_22_O_4_	0.76	Coumarin
27	7.42	297.14	Cryptotanshinone	296.4	C_19_H_20_O_3_	0.75	Diterpenoid
28	21.38	313.50	6-(1,1- dimethylallyl)-21-hydroxy-1-methylethyl)-2,3-dihydro-7 H-furo[3,2-G] chromen-7-one	314.4	C_19_H_22_O_4_	0.75	Coumarin

### ADMET properties

Nine compounds satisfied all five drug-likeness criteria - Lipinski, Ghose, Veber, Egan, and Muegge without any violations (Table 3). These compounds were (A) Usnic acid; (B) Anisomycin; (C) 5,6,2’-trimethoxyflavone (D) Cinchonine (E) 8,8-dimethyl-2,10-dioxo-9 H-pyrano[2,3-f]chromen-9-yl) (Z)-2-methylbut-2-enoate (F)7-[(2E,5E)-7-hydroxy-3,7-dimethylocta-2,5-dienoxy]chromen-2-one (G) 4,5-dihydroxy-4,5,6-trimethyl-2,8-dioxa-13-azatricyclo [8.5.1.013,16] hexadec-10-ene-3,7-dione (H) Cryptotanshinone (I) 6-(1,1-dimethyallyl)-2-(1-hydroxyl-1-methylethyl)-2,3-dihydro-7 H-furo[3,2-G]chromen-7-one. These compounds, which exhibited favorable physicochemical and drug-likeness properties, were subsequently subjected to ADMET (absorption, distribution, metabolism, excretion, and toxicity) profiling for further pharmacokinetic evaluation.

The Abbot Bioavailability Score (ABS) indicated a moderate probability of oral bioavailability (55–56%) for all compounds, except Cryptotanshinone, which exhibited a comparatively high score. Although these compounds satisfied major drug-likeness criteria, each presented at least one lead-likeness violation, indicating the need for structural optimization during lead development. Furthermore, compounds (E) 8,8-dimethyl-2,10-dioxo-9 H-pyrano[2,3-f]chromen-9-yl) (Z)-2-methylbut-2-enoate (F) 7-[(2E,5E)-7-hydroxy-3,7-dimethylocta-2,5-dienoxy]chromen-2-one, (G) 4,5-dihydroxy-4,5,6-trimethyl-2,8-dioxa-13-azatricyclo [8.5.1.013,16] hexadec-10-ene-3,7-dione (I) 6-(1,1-dimethyallyl)-2-(1-hydroxyl-1-methylethyl)-2,3-dihydro-7 H-furo[3,2-G]chromen-7-one triggered two BRENK alerts suggesting the presence of potentially problematic substructures that may compromise safety or synthetic feasibility.

The basic physicochemical properties of the selected compounds are summarized in Table 4. Molecular weights ranged from 265.3 to 344.32 g/mol, all below the 500 g/mol threshold, which supports compliance with Lipinski’s rule. The computed consensus Log P value (lipophilicity) varied between 0.9 and 3.87, indicating moderate lipophilicity favorable for oral absorption (Table 5). ADME analysis revealed favorable pharmacokinetic characteristics for all compounds (Table 6). All nine compounds demonstrated high gastrointestinal (GI) absorption, suggesting good oral bioavailability. Blood-brain barrier (BBB) permeability was predicted for several compounds, including (C) 5,6,2’-trimethoxyflavone; (D) Cinchonine; (F) 7-[(2E,5E)-7-hydroxy-3,7-dimethylocta-2,5-dienoxy] chromen-2-one; (G). 4,5-dihydroxy-4,5,6-trimethyl-2,8-dioxa-13-azatricyclo [8.5.1.013,16] hexadec-10-ene-3,7-dione (H) Cryptotanshinone; (I) 6-(1,1-dimethyallyl)-2-(1-hydroxyl-1-methylethyl)-2,3-dihydro-7 H-furo[3,2-G] chromen-7-one, indicating potential central nervous system activity. Among these, (C) Cinchonine and (H) Cryptotanshinone were also predicted to be P-gp substrates.

Regarding cytochrome P450 (CYP) inhibition, (A) Usnic acid and (B) Anisomycin exhibited no inhibitory activity against any of the CYP isoforms (Table 6a, b). Conversely, (C) 5,6,2’-trimethoxyflavone inhibited five CYP isoforms, while (H) Cryptotanshinone, and (I) 6-(1,1-dimethyallyl)-2-(1-hydroxyl-1-methylethyl)-2,3-dihydro-7 H-furo[3,2-G]chromen-7-one inhibited four isoforms. Compounds (E) 8,8-dimethyl-2,10-dioxo-9 H-pyrano[2,3-f]chromen-9-yl) (Z)-2-methylbut-2-enoate, (F) 7-[(2E,5E)-7-hydroxy-3,7-dimethylocta-2,5-dienoxy]chromen-2-one, and (G) 4,5-dihydroxy-4,5,6-trimethyl-2,8-dioxa-13-azatricyclo [8.5.1.013,16] hexadec-10-ene-3,7-dione 4[8.5.1.013,16] hexadec-10-ene-3,7-dione inhibited three isoforms each, whereas (D) Cinchonine inhibited one isoform.

Table 7 presents the predicted toxicity profile of bioactive compounds present in *D. meyeniana* extract. Compounds (C) 5,6,2’-trimethoxyflavone; (F) 7-[(2E,5E)-7-hydroxy-3,7-dimethylocta-2,5-dienoxy]chromen-2-one, (G) 4,5-dihydroxy-4,5,6-trimethyl-2,8-dioxa-13-azatricyclo [8.5.1.013,16] hexadec-10-ene-3,7-dione, and ( H) Cryptotanshinone exhibited LD_50_ values exceeding 2000 mg kg^− 1^, indicating low acute oral toxicity. Several compounds, including (B) Anisomycin, (C) 5,6,2’-trimethoxyflavone, (D) Cinchonine, (F) 7-[(2E,5E)-7-hydroxy-3,7-dimethylocta-2,5-dienoxy]chromen-2-one, and (G) 4,5-dihydroxy-4,5,6-trimethyl-2,8-dioxa-13-azatricyclo [8.5.1.013,16] hexadec-10-ene-3,7-dione, were predicted to be non-hepatotoxic, non-carcinogenic, non-cytotoxic, and non-mutagenic, suggesting a favorable safety profile. In contrast, (A) Usnic acid and (H) Cryptotanshinone were predicted to be carcinogenicity-active compounds. This prediction implies that both molecules may interact with molecular pathways involved in cell cycle regulation or DNA response^56^, potentially contributing to carcinogenic effects under certain exposure conditions. However, both compounds have also been reported to exhibit potent anticancer and pro-apoptotic activities, suggesting that their biological effects are dose-and context-dependent^57^. These findings highlight the necessity for experimental validations through toxicological assays to confirm their predicted carcinogenic potential. Additionally, (E) 8,8-dimethyl-2,10-dioxo-9 H-pyrano[2,3-f]chromen-9-yl) (Z)-2-methylbut-2-enoate and (I) 6-(1,1-dimethyallyl)-2-(1-hydroxyl-1-methylethyl)-2,3-dihydro-7 H-furo[3,2-G] chromen-7-one were predicted to exhibit immunotoxicity and mutagenicity, respectively, indicating potential structural concerns. For comparison, Erlotinib and Eprenetapopt (APR-246), a reference anticancer drug, displayed more pronounced toxicity, while Erlotinib showed cytotoxic activity, with other toxicological parameters remaining inactive.

**Table 3:** Predicted drug-likeness results of the volatile and non-volatile bioactive compounds of *D. meyeniana.*

Bioactive compounds	A	B	C	D	E	F	G	H	I	J	K
ADME properties											
Lipinski # violations	Yes; 0	Yes; 0	Yes; 0	Yes; 0	Yes; 0	Yes; 0	Yes; 0	Yes; 0	Yes; 0	Yes; 0	Yes; 0
Ghose # violations	Yes; 0	Yes; 0	Yes; 0	Yes; 0	Yes; 0	Yes; 0	Yes; 0	Yes; 0	Yes; 0	Yes; 0	No; 1
Veber # violations	Yes; 0	Yes; 0	Yes; 0	Yes; 0	Yes; 0	Yes; 0	Yes;0	Yes; 0	Yes; 0	Yes; 0	Yes; 0
Egan # violations	Yes; 0	Yes; 0	Yes; 0	Yes; 0	Yes; 0	Yes; 0	Yes; 0	Yes; 0	Yes; 0	Yes; 0	Yes; 0
Muegge # violations	Yes; 0	Yes; 0	Yes; 0	Yes; 0	Yes;0	Yes; 0	Yes; 0	Yes; 0	Yes; 0	Yes; 0	No; 1
Bioavailability score	0.56	0.55	0.55	0.55	0.55	0.55	0.55	0.85	0.55	0.55	0.55
Pan assay interference compounds (PAINS) # alerts	0	0	0	0	0	0	0	2	0	0	0
Brenk # alerts	1	0	0	1	2	2	2	1	2	1	0
Lead likeness # violations	0	0	0	0	0	1	1	1	1	2	1
Synthetic accessibility	4.19	3.14	3.46	4.18	4.09	3.57	3.57	4.11	4	3.19	3.56

(A) Usnic acid (B) Anisomycin (C) 5,6,2’-Trimethoxyflavone (D) Cinchonine (E) 8,8-dimethyl-2,10-dioxo-9 H-pyrano[2,3-f]chromen-9-yl) (Z)-2-methylbut-2-enoate (F) 7-[(2E,5E)-7-hydroxy-3,7-dimethylocta-2,5-dienoxy]chromen-2-one (G) 4,5-dihydroxy-4,5,6-trimethyl-2,8-dioxa-13-azatricyclo[8.5.1.013,16]hexadec-10-ene-3,7-dione (H) Cryptotanshinone (I)6-(1,1-dimethylallyl)-2-(1-hydroxy-1-methylethl)-2,3-Dihydro-7 H-furo[3,2-G]chromen-7-one (J) Erlotinib (K) Eprenetapopt (APR-246).

**Table 4:** Physiochemical properties and computational descriptors of the volatile and non-volatile bioactive compounds of *D. meyeniana.*

Bioactive compounds	A	B	C	D	E	F	G	H	I	J	K
ADME properties											
Size (MW) g/mol	344.32	265.3	312.32	294.39	342.34	314.38	314.38	296.36	314.38	393.44	199.25
Lipophilicity XLOGP3) Solubility Log S (ESOL)	2.88	0.9	3.09	2.68	3.2	3.52	3.52	3.8	3.87	3.31	-0.09
Solubility Log S (ESOL)	-3.83	-1.96	-3.97	-3.49	-9.55	-3.93	-3.93	-4.27	-4.35	-4.11	-0.82
Saturation Fraction Csp3	0.33	0.5	0.17	0.42	0.32	0.32	0.32	0.47	0.42	0.27	0.9
Flexibility (# rotable bonds)	2	5	4	3	3	6	6	0	3	10	3
Num. H-bond acceptors	7	5	5	3	6	4	4	3	4	6	4
Num. H-bond donors	2	2	0	1	0	1	1	0	1	1	1
Molar refractivity	86.54	87.4	87.4	93.24	91.55	92.49	92.49	85.13	91.05	111.4	55.25

(A) Usnic acid (B) Anisomycin (C) 5,6,2’-Trimethoxyflavone (D) Cinchonine (E) 8,8-dimethyl-2,10-dioxo-9 H-pyrano[2,3-f]chromen-9-yl) (Z)-2-methylbut-2-enoate (F) 7-[(2E,5E)-7-hydroxy-3,7-dimethylocta-2,5-dienoxy]chromen-2-one (G) 4,5-dihydroxy-4,5,6-trimethyl-2,8-dioxa-13-azatricyclo[8.5.1.013,16]hexadec-10-ene-3,7-dione (H) Cryptotanshinone (I)6-(1,1-dimethylallyl)-2-(1-hydroxy-1-methylethl)-2,3-Dihydro-7 H-furo[3,2-G]chromen-7-one (J) Erlotinib (K) Eprenetapopt (APR-246).

**Table 5:** Lipophilicity results of the volatile and non-volatile bioactive compounds of *D. meyeniana.*

Bioactive compounds	A	B	C	D	E	F	G	H	I	J	K
ADME properties											
Polarity TPSA	117.97	67.79	57.9	36.36	82.81	59.67	59.67	43.37	59.67	74.73	49.77
XLOGP3	2.88	0.9	3.09	2.68	3.2	3.52	3.52	3.8	3.87	3.31	-0.09
ILOGP	0.85	2.34	3.1	3.07	2.71	3.6	3.6	2.81	3.53	3.67	2.02
WLOGP	1.5	0.12	3.49	2.46	3.02	3.84	3.84	3.44	3.33	3.48	-0.72
MLOGP	-0.52	0.71	1.25	2.59	1.76	2.66	2.66	2.66	2.74	1.48	-0.35
SILICOS-IT	2.8	1.4	4.08	3.08	3.76	4.37	4.37	4.37	4.48	4.06	0.84
Lipophilicity average	1.5	1.1	3	2.78	2.89	3.6	3.6	3.6	3.59	3.2	0.34

(A) Usnic acid (B) Anisomycin (C) 5,6,2’-Trimethoxyflavone (D) Cinchonine (E) 8,8-dimethyl-2,10-dioxo-9 H-pyrano[2,3-f]chromen-9-yl) (Z)-2-methylbut-2-enoate (F) 7-[(2E,5E)-7-hydroxy-3,7-dimethylocta-2,5-dienoxy]chromen-2-one (G) 4,5-dihydroxy-4,5,6-trimethyl-2,8-dioxa-13-azatricyclo[8.5.1.013,16]hexadec-10-ene-3,7-dione (H) Cryptotanshinone (I)6-(1,1-dimethylallyl)-2-(1-hydroxy-1-methylethl)-2,3-Dihydro-7 H-furo[3,2-G]chromen-7-one (J) Erlotinib (K) Eprenetapopt (APR-246).

**Table 6:** Predicted pharmacokinetics results of the volatile and non-volatile bioactive compounds of *D. meyeniana.*

Bioactive compounds	A	B	C	D	E	F	G	H	I	J	K
ADME properties											
Gastrointestinal (GI) absorption	High	High	High	High	High	High	High	High	High	High	High
Blood-brain barrier (BBB) permeability	No	No	Yes	Yes	No	Yes	Yes	Yes	Yes	Yes	No
P-gp substrate	No	No	No	Yes	No	No	No	Yes	No	No	No
CYP1A2 inhibitor	No	No	Yes	No	No	Yes	Yes	Yes	Yes	Yes	No
CYP2C19 inhibitor	No	No	Yes	No	Yes	Yes	Yes	Yes	Yes	Yes	No
CYP2C9 inhibitor	No	No	Yes	No	Yes	Yes	Yes	Yes	Yes	Yes	No
CYP2D6 inhibitor	No	No	Yes	Yes	No	No	No	No	Yes	Yes	No
CYPD3A4 inhibitor	No	No	Yes	No	Yes	No	No	Yes	No	Yes	No
Log Kp (skin permeation) cm/s	-6.36	-7.28	-6.01	-6.19	-6.12	-5.72	-5.72	-5.41	-5.47	-6.35	-7.58

(A) Usnic acid (B) Anisomycin (C) 5,6,2’-Trimethoxyflavone (D) Cinchonine (E) 8,8-dimethyl-2,10-dioxo-9 H-pyrano[2,3-f]chromen-9-yl) (Z)-2-methylbut-2-enoate (F) 7-[(2E,5E)-7-hydroxy-3,7-dimethylocta-2,5-dienoxy]chromen-2-one (G) 4,5-dihydroxy-4,5,6-trimethyl-2,8-dioxa-13-azatricyclo[8.5.1.013,16]hexadec-10-ene-3,7-dione (H) Cryptotanshinone (I)6-(1,1-dimethylallyl)-2-(1-hydroxy-1-methylethl)-2,3-Dihydro-7 H-furo[3,2-G]chromen-7-one (J) Erlotinib (K) Eprenetapopt (APR-246).

**Table 7:** Toxicological properties of selected bioactive compounds of *D. meyeniana*.

Code	Bioactive compounds	Hepatotoxicity		Carcinogenicity		Cytotoxicity		Immunotoxicity		Mutagenicity		Predicted LD50 (mg kg-^1^)	Toxicity class
		Pr	Pb	Pr	Pb	Pr	Pb	Pr	Pb	Pr	Pb		
A	Usnic acid	Inactive	0.61	Active	0.57	Inactive	0.72	Inactive	0.51	Inactive	0.89	995	4
B	Anisomycin	Inactive	0.89	Inactive	0.73	Inactive	0.63	Inactive	0.66	Inactive	0.78	72	3
C	5,6,2’-trimethoxyflavone	Inactive	0.69	Inactive	0.53	Inactive	0.82	Inactive	0.81	Inactive	0.69	4000	5
D	Cinchonine	Inactive	0.93	Inactive	0.67	Inactive	0.8	Inactive	0.6	Inactive	0.68	720	4
E	8,8-dimethyl-2,10-dioxo-9 H-pyrano [2,3- f] chromen-9-yl) (Z)-2-methylbut-2-enoate	Inactive	0.63	Inactive	0.54	Inactive	0.67	Active	0.9	Inactive	0.54	832	4
F	7-[(2E,5E)-7-hydroxy-3,7-dimethylocta-2,5- dienoxy] chromen-2-one	Inactive	0.78	Inactive	0.56	Inactive	0.78	Inactive	0.89	Inactive	0.54	3200	5
G	4,5-dihydroxy-4,5,6-trimethyl-2,8-dioxa-13-azatricyclo [8.5.1.013,16] hexadec-10-ene-3,7-dione	Inactive	0.78	Inactive	0.56	Inactive	0.78	Inactive	0.89	Inactive	0.54	3200	5
H	Cryptotanshinone	Inactive	0.74	Active	0.51	Inactive	0.78	Active	0.96	Inactive	0.79	8000	6
I	6-(1,1-dimethylallyl)-2-(1-hydroxy-1- methylethyl)-2,3-dihydro-7 H-Furo[3,2-G] chromen − 7- one	Inactive	0.69	Inactive	0.58	Inactive	0.7	Inactive	0.98	Active	0.82	1500	4
J	Erlotinib	Active	0.78	Inactive	0.51	Active	0.75	Active	0.91	Active	0.55	125	3
K	Eprenetapopt (APR-246)	Inactive	0.93	Inactive	0.52	Inactive	0.75	Inactive	0.97	Inactive	0.73	900	4

*Pr: prediction; Pb: probability.

**Fig. 1:**
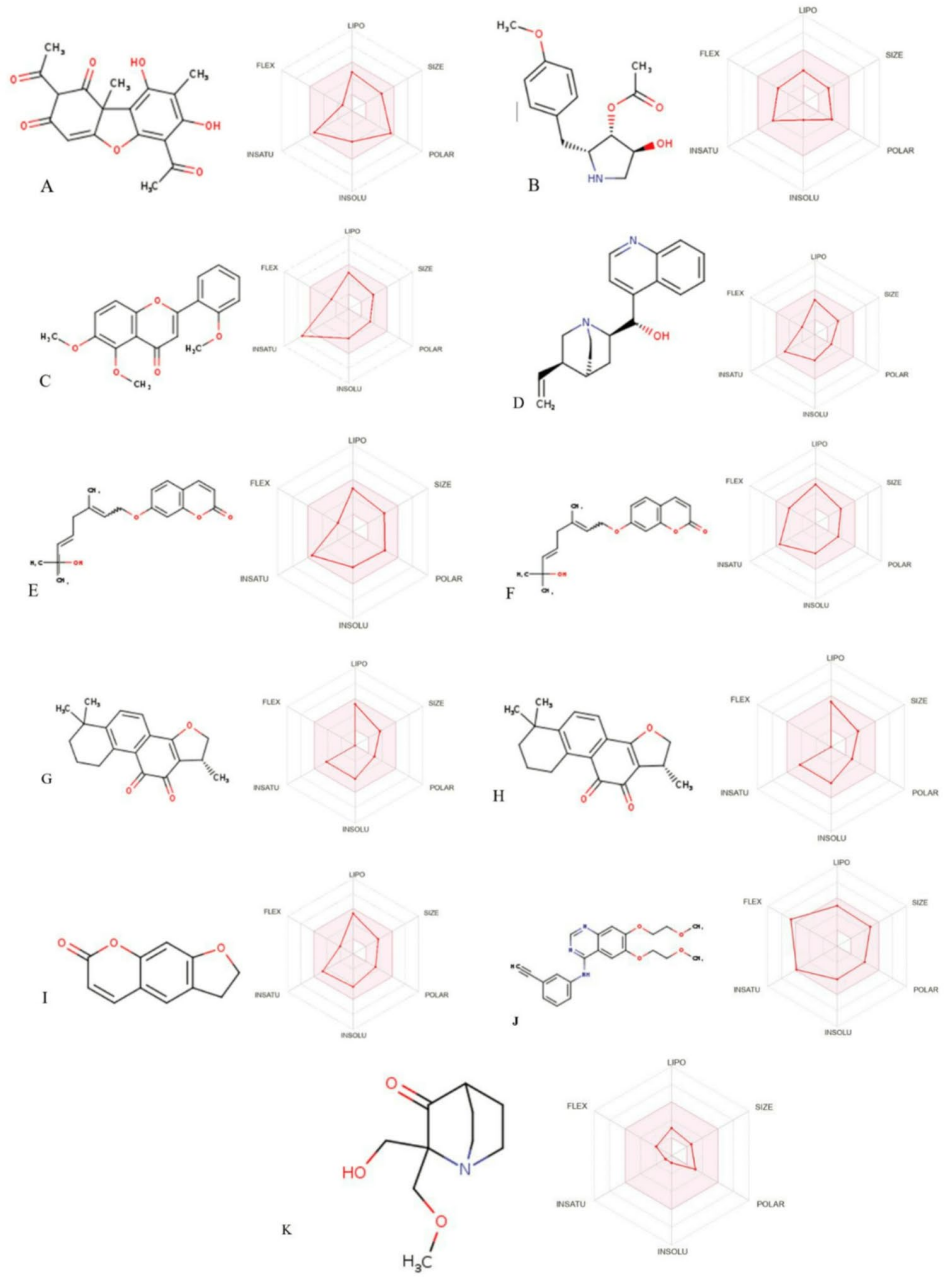
Bioavailability radar of the bioactive compounds of *D. meyeniana* **(A)** Usnic acid; **(B)** Anisomycin; **(C)** 5,6,2’-trimethoxyflavone **(D)** Cinchonine **(E)** 8,8-dimethyl-2,10-dioxo-9 H-pyrano[2,3-f]chromen-9-yl) (Z)-2-methylbut-2-enoate **(F)**7-[(2E,5E)-7-hydroxy-3,7-dimethylocta-2,5-dienoxy]chromen-2-one (**G)** 4,5-dihydroxy-4,5,6-trimethyl-2,8-dioxa-13-azatricyclo [8.5.1.013,16] hexadec-10-ene-3,7-dione **(H)** Cryptotanshinone **(I)** 6-(1,1-dimethyallyl)-2-(1-hydroxyl-1-methylethyl)-2,3-dihydro-7 H-furo[3,2-G]chromen-7-one and reference drugs (**J**) Erlotinib (**K**) Eprenetapopt (APR-246).

The bioavailability radar plot (Fig. 1) illustrates the drug-likeness profiles of the bioactive compounds, evaluating six key physicochemical properties: lipophilicity, size, polarity, solubility, flexibility, and saturation. Eight bioactive compounds namely (A) Usnic acid; (B) Anisomycin; ( D) Cinchonine; (E) 8,8-dimethyl-2,10-dioxo-9 H-pyrano[2,3-f]chromen-9-yl) (Z)-2-methylbut-2-enoate; (F) 7-[(2E,5E)-7-hydroxy-3,7-dimethylocta-2,5-dienoxy]chromen-2-one; (G) 4,5-dihydroxy-4,5,6-trimethyl-2,8-dioxa-13-azatricyclo [8.5.1.013,16] hexadec-10-ene-3,7-dione (H) Cryptotanshinone and (I) 6-(1,1-dimethyallyl)-2-(1-hydroxyl-1-methylethyl)-2,3-dihydro-7 H-furo[3,2-G]chromen-7-one fully occupy the pink area of the radar, indicating compliance with optimal ranges for oral bioavailability. In contrast, (C) 5,6,2’-trimethoxyflavone did not meet the saturation criterion (sp3 carbon fraction, greater than 0.25).

### Molecular docking analysis

The present study aimed to identify bioactive compounds from *D. meyeniana* capable of binding to and inhibiting key cancer-related proteins through molecular docking analysis. Four cancer-related proteins- EGFR, p53, MMP7, and CDK8/Cyclin C were retrieved from the Research Collaboratory of Structural Bioinformatics Protein Data Bank (RCSB PDB), while nine compounds previously identified in *D. meyeniana* were obtained in PubChem. Prior to docking, all protein structures were pre-processed to remove crystallographic water molecules, heteroatoms, and reductant chains. Missing side chains and hydrogen atoms were added to ensure proper geometry, followed by energy minimization to relieve local steric strains. The structural integrity and stereochemical quality of the processed proteins were subsequently assessed using Ramachandran Protein analysis.

**Fig. 2:**
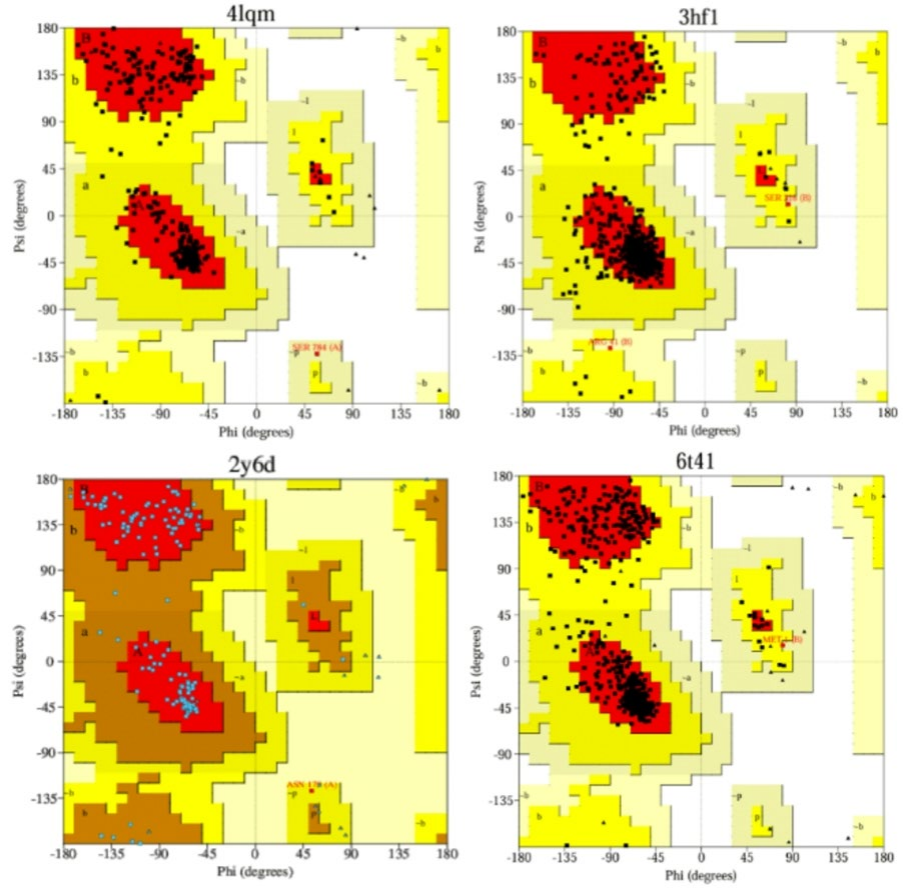
Ramchandran plot analysis of the four cancer–related proteins EGFR (CID: 4lqm), p53 (3hf1), MMP7 (2y6d), and CDK8/Cyclin C (6t41).

As shown in Fig. 2, the Ramachandran plots revealed that the majority of residues for all target proteins occupied the most favored and additionally allowed regions, with only a minimal fraction (< 2%) located in disallowed regions. This distribution indicates that the backbone dihedral angles (φ and ψ) of the protein residues are stereochemically favorable, confirming the structural reliability and suitability of the protein models for molecular docking. The high percentage of residues in the favored region further supports the absence of major conformational distortions or modeling errors, thereby validating the quality of the protein structures employed in subsequent docking simulations.

In structure-based drug discovery, molecular docking serves as a pivotal computational approach to identify potential ligand hits against specific protein targets. This method predicts the optimal orientation, position, and conformation of the ligands within the protein’s active site, thereby elucidating the nature and strength of their binding interaction and affinities. In the present study, molecular docking was performed using AutoDock Vina integrated within PyRx, which utilizes a grid-based algorithm to estimate binding energies and generate possible ligand poses. A total of nine bioactive compounds were docked against cancer-related targets to assess their potential inhibitory interaction. Table 8 summarizes the binding interactions of *D. meyeniana* compounds that satisfied all drug-likeness criteria. These selected compounds include: (A) Usnic acid; (B) Anisomycin; (C) 5,6,2’-trimethoxyflavone (D) Cinchonine; (E) 8,8-dimethyl-2,10-dioxo-9 H-pyrano[2,3-f]chromen-9-yl) (Z)-2-methylbut-2-enoate; (F) 7-[(2E,5E)-7-hydroxy-3,7-dimethylocta-2,5-dienoxy]chromen-2-one; (G) 4,5-dihydroxy-4,5,6-trimethyl-2,8-dioxa-13-azatricyclo [8.5.1.013,16] hexadec-10-ene-3,7-dione (H) Cryptotanshinone; (I) 6-(1,1-dimethyallyl)-2-(1-hydroxyl-1-methylethyl)-2,3-dihydro-7 H-furo[3,2-G]chromen-7-one Molecular docking performed against four key cancer-related targets: epidermal growth factor receptor (EGFR; PD ID: 4lqm), tumor suppressor protein p53 (PD ID: 3hf1), matrix metalloproteinase-7 (MMP7; PD ID: 2y6d), and cyclin-dependent kinase 8/ Cyclin C (CDK8/Cyclin C; PD ID: 6t41). Erlotinib, a known epidermal growth factor receptor (EGFR) inhibitor, is clinically used in the treatment of certain types of non-small cell lung cancer^37^. Eprenetapopt (APR-246 ), a first-in-class small molecule that reactivates mutant p53, induces apoptosis, has been designated as a Fast Track and Orphan drug by the FDA for a certain indication^38^. Both Erlotinib and Eprenetapopt, recognized as FDA-approved chemotherapeutic agents for various carcinomas^58,59^ were employed as a reference compound for comparative analysis.

Among the nine compounds, four screened exhibited strong binding affinity toward EGFR. Cryptotanshinone (H) showed the most favorable binding energy (-8.3 kcal/mol), followed by (A) Usnic acid and (C) 5,6,2’-trimethoxyflavone (-7.9 kcal/mol); (E) 8,8-dimethyl-2,10-dioxo-9 H-pyrano[2,3-f]chromen-9-yl) (Z)-2-methylbut-2-enoate (-7.3 kcal/mol); (F) 7-[(2E,5E)-7-hydroxy-3,7-dimethylocta-2,5-dienoxy]chromen-2-one (-7.2 kcal/mol) and (I) 6-(1,1-dimethyallyl)-2-(1-hydroxyl-1-methylethyl)-2,3-dihydro-7 H-furo[3,2-G]chromen-7-one (-7.2 kcal/mol). These values were comparable to those of the reference inhibitor Erlotinib, an FDA-approved EGFR-targeting drug used in non-small cell lung carcinoma (-9.0 3 kcal/mol), indicating promising inhibitory potential. For matrix metalloproteinase-7 (MMP-7), the compounds exhibited binding affinities from − 6.3 to -8.7 kcal/mol, with Cryptotanshinone again displaying the strongest interaction (-8.7 kcal/mol). In the case of cyclin-dependent kinase 8 (CDK8/Cyclin C), all the ligands demonstrated high binding affinities, with energies between − 7.4 and − 9.8 kcal/mol. Cryptotanshinone remained the top-performing compound (-9.8 kcal/m), showing a binding energy comparable to the binding energy to the reference chemotherapeutic drug, Erlotinib (-10.5 kcal/mol).

**Fig. 3:**
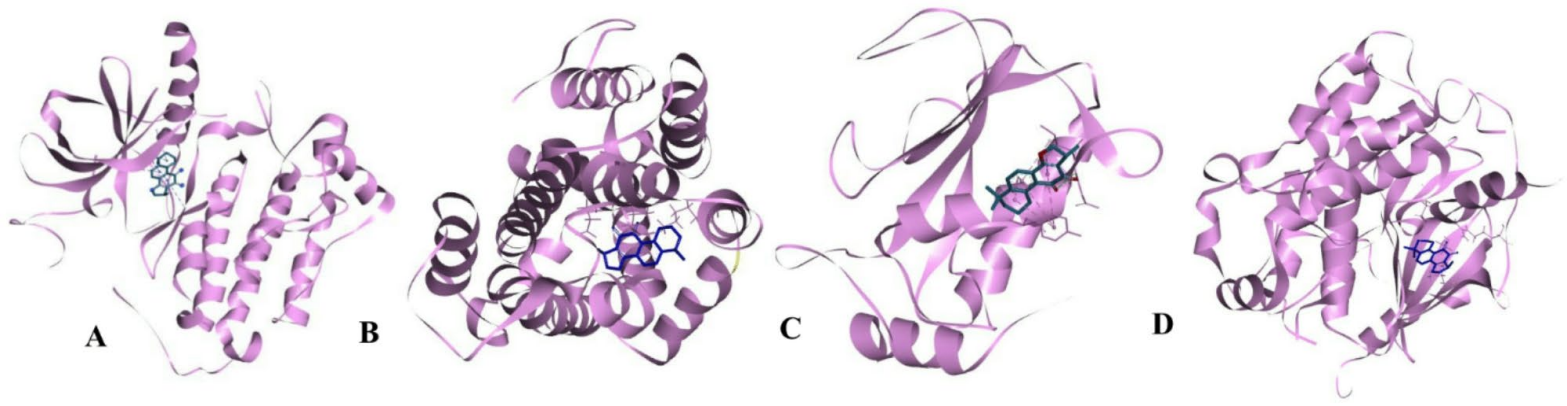
Three-dimensional crystal structure of cancer-related proteins with co-crystallized inhibitor. (**A**) EGFR-cryptotanshinone (**B**), p53-Cryptotanshinone (**C**), MMP7 Cryptotanshinone (**D**), CDK8/ Cyclin C – Cryptotanshinone.

Ligand efficiency (LE) reflects ligand’s ability to achieve favorable binding interactions relative to tis molecular size when bound to the target receptor (Table 8) A threshold value of LE ≥ 0.3 kcal/mol per heavy atom is generally considered the benchmark for efficient binding^60^. In this study, all the compounds exhibited LE values above 0.3 except 8,8-dimethyl-2,10-dioxo-9 H-pyrano[2,3-f]chromen-9-yl) (Z)-2-methylbut-2-enoate and 4,5-dihydroxy-4,5,6-trimethyl-2,8-dioxa-13-azatricyclo [8.5.1.013,16] hexadec-10-ene-3,7-dione lower efficiencies. Usnic acid also displayed LE values below 0.3 against cancer-related targets (p53, MMP7 and CDK8/ Cyclin C), suggesting reduced efficiency compared to other ligands. The predicted inhibition constant (Ki) of the bioactive compounds ranged from 0.42 to 9.01µM, indicating moderate to strong inhibitor potential toward the cancer-related proteins. Notably, compounds with Ki values below 1 µM demonstrated particularly strong binding affinities, comparable to those of the reference drug (Table 8). These results suggest that several compounds possess favorable binding efficiencies and inhibitory characteristics suitable for further optimization as potential anticancer agents.

**Table 8:** Binding energy of bioactive compounds with target cancer-related protein receptors.

Code	Ligand	EGF R(PD ID: 4lqm)				p53 (PD ID: 3hf1)				MMP7 (PD ID: 2y6d)				CDK8/Cyclin C (PD ID: 6t41)			
		Binding Energy	Ligand Efficiency	Inhibitory Constant (µM)	No of H Bonds	Binding Energy	Ligand Efficiency	Inhibitory Constant (µM)	No of H Bonds	Binding Energy	Ligand Efficiency	Inhibitory Constant (µM)	No of H Bonds	Binding Energy	Ligand Efficiency	Inhibitory Constant (µM)	No of H Bonds
A	Usnic acid (CID: 5646)	-7.9	-0.25	1.59	2	-8.3	-0.24	8.10	4	-8.6	-0.23	4.88	1	-9.6	-0.21	9.01	2
B	Anisomycin (CID 253602)	-6.9	-0.72	8.63	0	-7.3	-0.68	4.39	3	-6.3	-0.79	2.38	1	-7.7	-0.64	2.23	1
C	5,6,2’-trimethoxyflavone (CID: 14484690)	-7.9	-0.51	1.59	0	-7.7	-0.52	2.23	1	-8.0	-0.50	1.34	0	-7.8	-0.51	1.88	1
D	Cinchonine (CID: 90454)	-7.2	-0.50	5.20	1	-7.5	-0.40	3.13	1	-8.2	-0.36	9.60	1	-8.2	-0.37	9.60	0
E	8,8-dimethyl-2,10-dioxo-9 H-pyrano[2,3-f] chromen-9-yl) (Z)-2-methylbut-2-enoate (CID: 51136479)	-7.3	-0.29	4.39	1	-7.7	-0.39	2.23	2	-7.3	-0.41	4.39	0	-7.6	-0.39	2.64	0
F	7-[(2E,5E)-7-hydroxy-3,7-dimethylocta-2,5-dienoxy] chromen-2-one (CID: 25763650)	-7.2	-0.83	5.20	0	-7.3	-0.82	4.39	1	-7.2	-0.83	5.20	1	-7.5	-0.8	3.13	0
G	4,5-dihydroxy-4,5,6-trimethyl-2,8-dioxa-13-azatricyclo [8.5.1.013,16] hexadec-10-ene-3,7-dione (56776345)	-7.2	-0.08	5.20	0	-7.6	-0.78	2.64	0	-6.9	-0.87	8.63	0	-7.4	-0.81	3.71	0
H	Cryptotanshinone (CID: 160254)	-8.8	-0.36	3.48	1	-8.7	-0.34	4.12	0	-8.7	-0.34	4.12	0	-9.8	-0.30	6.43	00
I	6-(1,1-dimethyallyl)-2-(1-hydroxyl-1-methylethyl)-2,3-dihydro-7 H-furo[3,2-G] chromen-7-one (CID: 151467)	-7.2	-0.31	5.20	1	-6.9	-0.43	8.63	0	-8.4	-0.36	6.84	0	-7.7	-0.39	2.23	1
J	Erlotinib (176870)	-9.0	-0.72	-1.11	1	-7.6	-1.32	2.64	1	-8.9	-1.12	2.94	0	-10.5	-0.95	1.97	1
K	Eprenetapopt (APR-246) CID: 52,918,385 (reference drug)	-4.9	-0.61	1.6	0	-5.7	-0.53	6.56	0	-5.4	-0.55	0.00	1	-5.8	-0.52	5.54	0

**Fig. 4:**
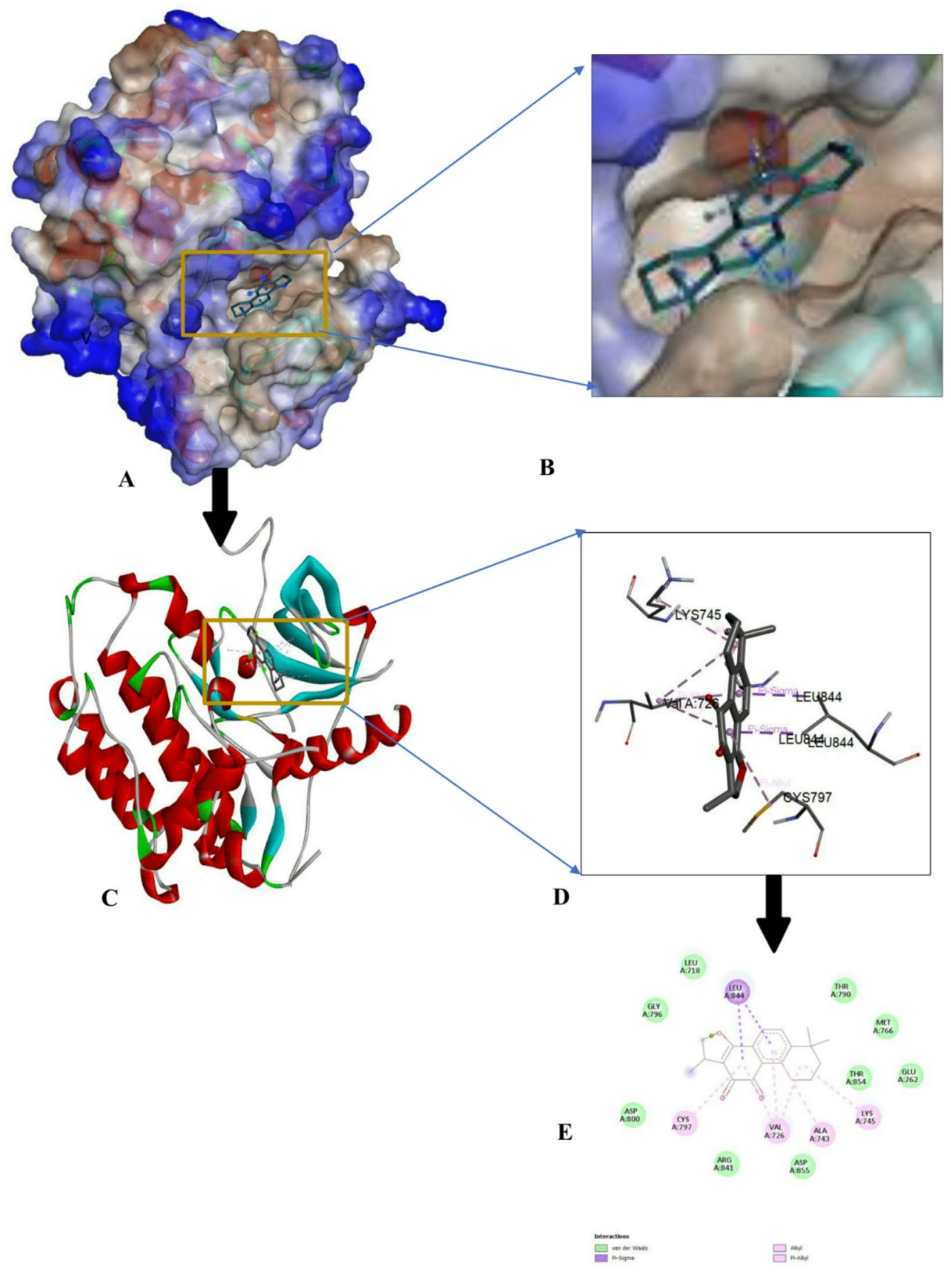
Docking interaction of Cryptotanshinone with EGFR (4LQM) (**A**) Molecular surface of the docked complex. (**B**) The 3D hydrophobicity surface plot at the binding site. (**C**) Overall ribbon structure. (**D**) Ligand interaction with target protein. (**E**) 2-D interaction of the complex.

**Fig. 5:**
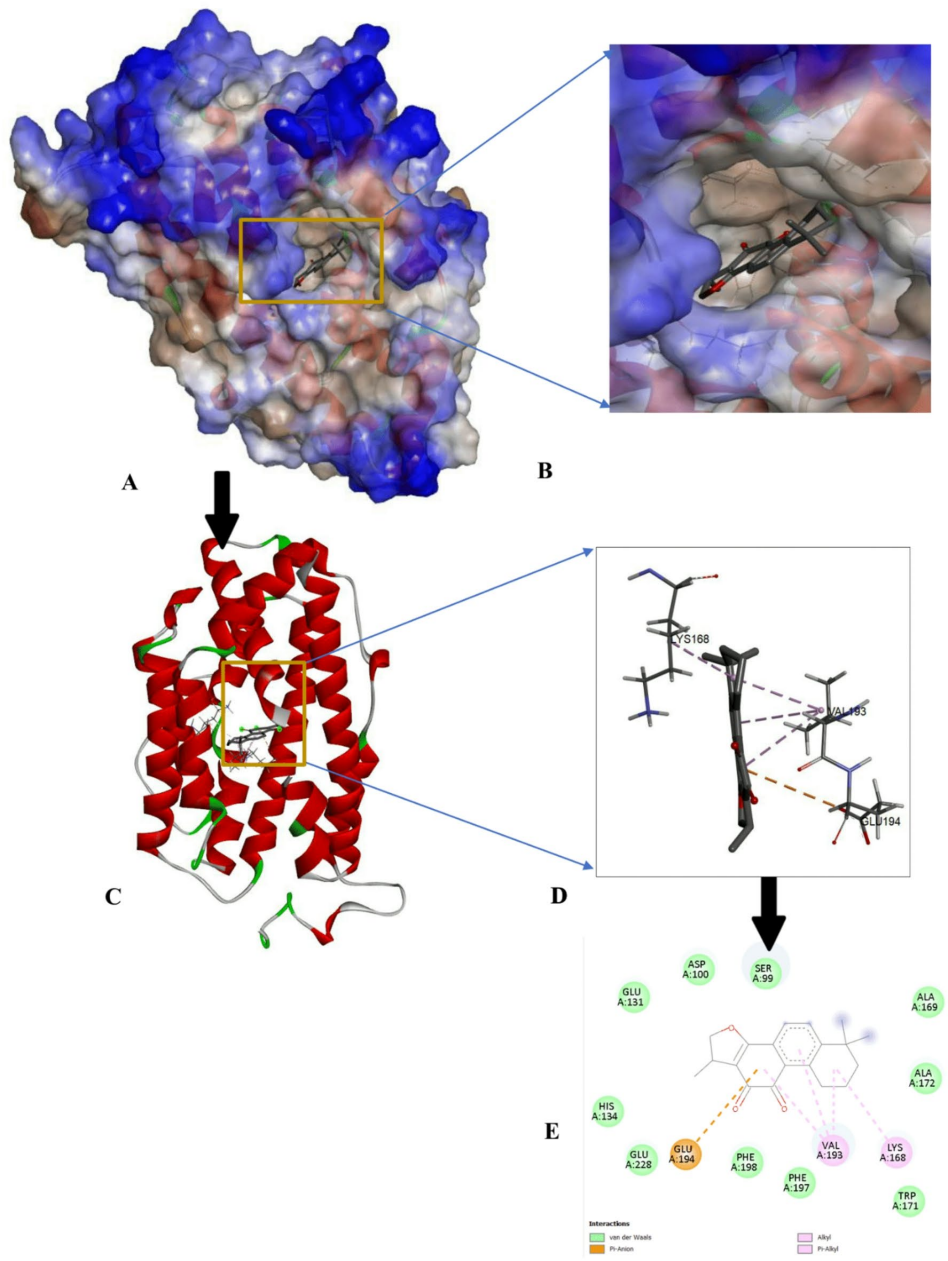
Docking interaction of Cryptotanshinone with p53 (3HF1). (**A**) Molecular surface of the docked complex. (**B**) The 3D hydrophobicity surface plot at the binding site. (**C**) Overall ribbon structure. (**D**) Ligand interaction with target protein. (**E**) 2-D interaction of the complex.

**Fig. 6:**
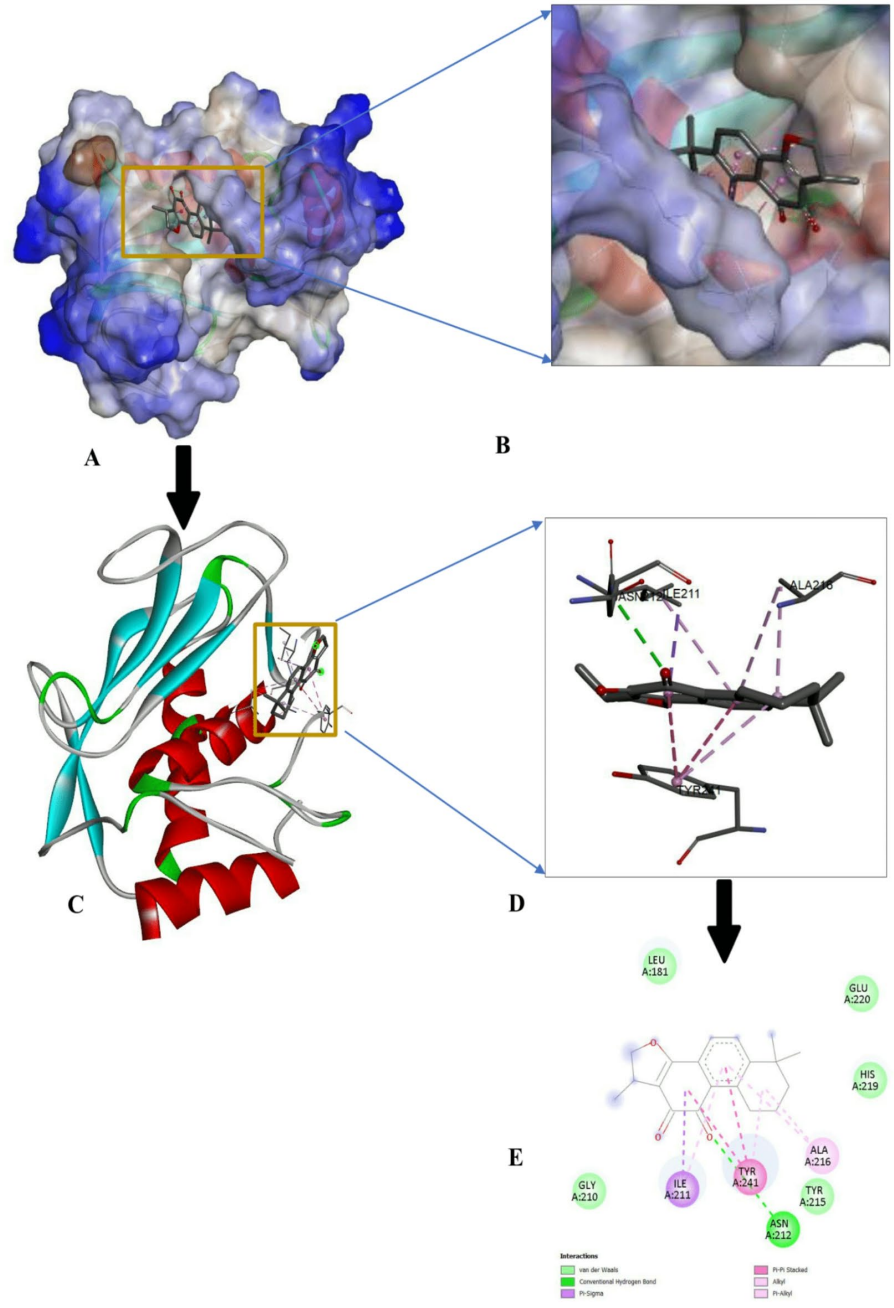
Docking interaction of Cryptotanshinone with MMP7 (2Y6D). (**A**) Molecular surface of the docked complex. (**B**) 3D hydrophobicity surface plot at the binding site. (**C**) Overall ribbon structure. (**D**) Ligand interaction with target protein. (**E**) 2-D interaction of the complex.

**Fig. 7:**
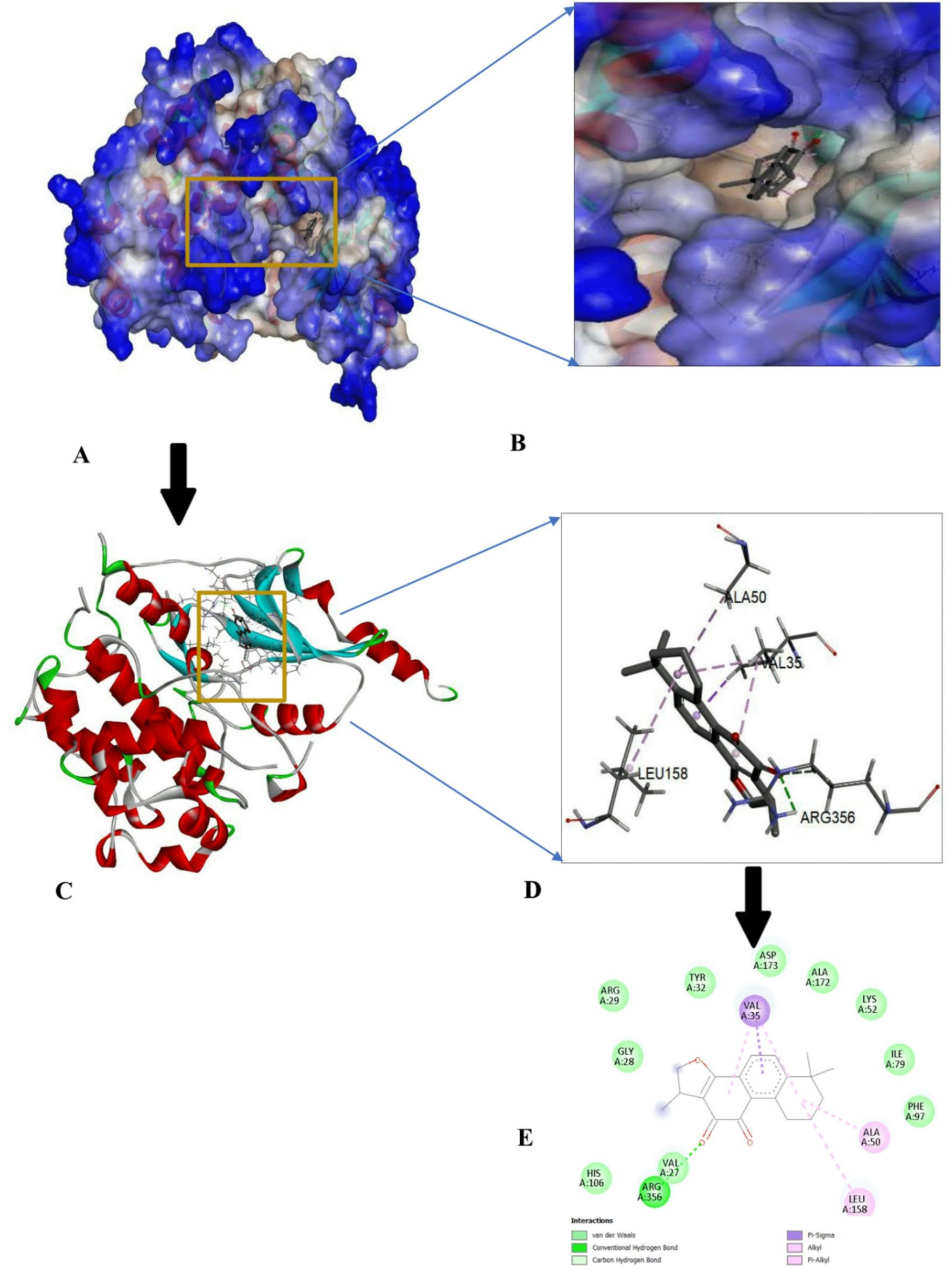
Docking interaction of Cryptotanshinone with CDK8/Cyclin C 6T41). (**A**) Molecular surface of the docked complex. (**B**) The 3D hydrophobicity surface plot at the binding site. (**C**) Overall ribbon structure. (**D**) Ligand interaction with target protein. (**E**) 2-D interaction of the complex.

The protein-ligand interactions of the top-ranked bioactive compounds and with each target protein are illustrated in Fig. 3, highlighting key residues and non-covalent interactions that contribute to complex stability. The compound with the highest binding affinities was further analyzed and visualized using Discovery Studio (https://discover.3ds.com/discovery-studio-visualizer-download). Among the screened ligands, Cryptotanshinone consistently exhibited the most favorable, highest binding energies to all cancer-related targets with binding affinities of -8.8 kcal/mol for EGFR, -8.7 kcal/mol for p53 and MMP7, and − 9.8 kcal/mol for CDK8/Cyclin C (Figs. 4, 5, 6 and 7). These strong interactions suggest that cryptotanshinone forms stable protein-ligand complexes, potentially contributing to this inhibitory activity against multiple cancer-associated proteins.

The key molecular interaction observed during the visualization include ℼ-sigma interaction, which facilitate ligand interaction within the receptor binding sites; hydrogen bonds, which contributes to complex stability; that helps ligand to intercalate at receptor binding sites; hydrogen provides stability to the complex; van der Waals forces, which enhance ligand binding through short- range attractions between non-polar regions, ensuring shape complementary between the ligand and receptor, and ℼ- alky interaction, which influence the ligands dipole moment and orientation within the binding pocket. For Cryptotanshinone interacting with the epidermal growth factor receptor (EGFR) (Fig. 4E), the complex was stabilized by van der Waals interactions with Thr 854, Asp 855, Arg 841, Asp 800, Gly 796, Leu 718, Thr 790, Met 766, and Glu 762; and an alkyl interaction with Cys 797, Val 726, Ala 743, and Lys 745; and ℼ sigma Leu 844.

In its interaction with tumor suppressor protein p53 (Fig. 5E), Cryptotanshinone formed van der Waals interactions with Phe 197, Glu 228, Phe 198, His 134, Glu 131, Asp 100, Ser 99, Ala 169, Ala 172, and Trp 171; a ℼ-cation bond interaction with Glu 194; a ℼ-alkyl with Val 193 and Lys 168. For matrix metalloproteinase-7 (MMP7; PD ID: 2y6d) complex (Fig. 6E), Cryptotanshinone established a conventional hydrogen bond with Asn 212, van der Waals interaction with Gly 210, Leu 181, Glu 220, His 219, and Tyr 215, a ℼ-sigma bond with Ile 211, a ℼ-ℼ-stacked interaction with Tyr 241; and addition of hydrophobic alkyl (ℼ-alkyl) interactions involving Ala 216. Finally, in its interaction with cyclin-dependent kinase 8/ Cyclin C (CDK8/Cyclin C; PD ID: 6t41) (Fig. 7E). Cryptotanshinone formed a conventional hydrogen bond with Arg 356; van der Waals interactions with His 106, Gly 28, Arg 29, Tyr 32, Asp 173, Ala 172, Lys 52, Ile 79, and Phe 97; a ℼ-sigma with Val 35; and ℼ-alkyl with Ileu 158 and Ala 50. Overall, Cryptotanshinone exhibited a strong and diverse interaction profile, particularly with MMP7 and CDK8/Cyclin C, characterized by a greater number of hydrogen bonds and extensive hydrophobic contacts. These features are consistent with their higher binding affinities and highlight Cryptotanshinone potential as a promising therapeutic candidate.

### Molecular dynamics simulation

**Fig. 8:**
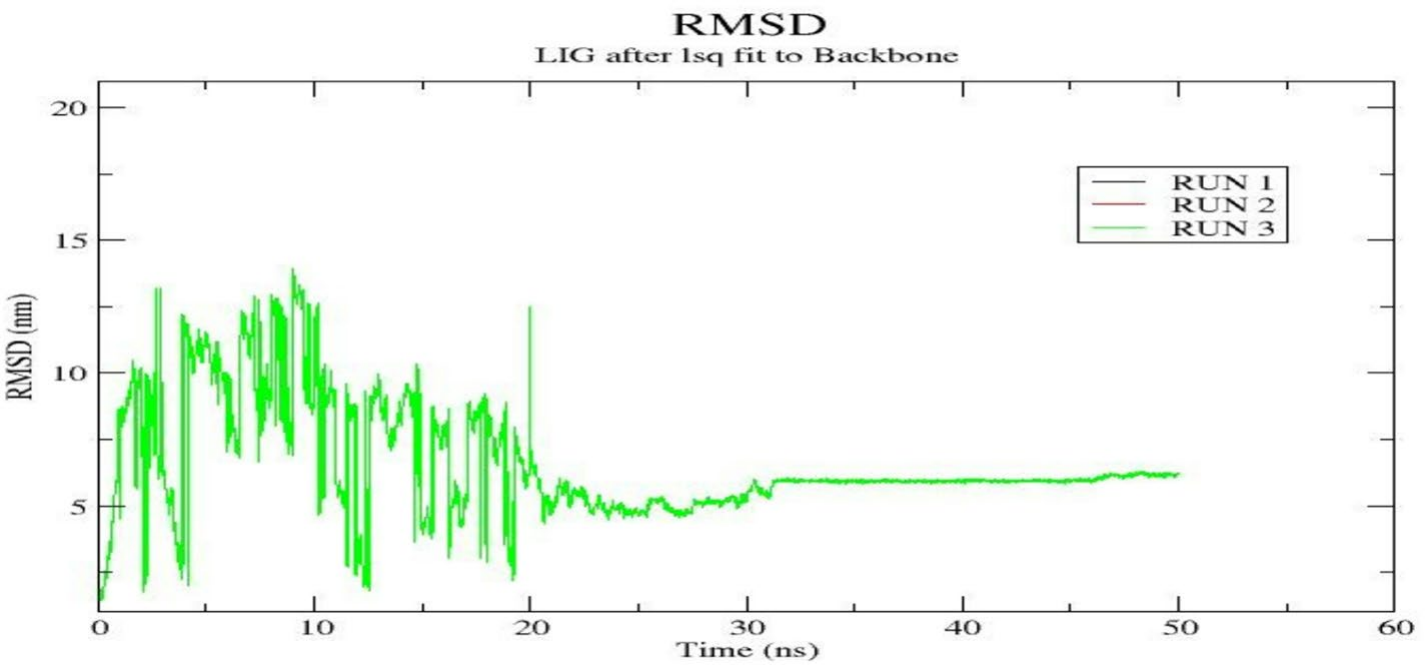
The RMSD of ligand atoms relative to EGFR backbone atoms in triplicate MD simulation runs for the EGFR- Cryptotanshinone complexes.

The EGFR- Cryptotanshinone complex was further subjected to molecular dynamics (MD) simulation to evaluate its conformational stability and dynamic behavior under simulated physiological conditions. The structural stability and flexibility of the complex were assessed using four key parameters: root mean square deviation (RMSD), root mean square fluctuation (RMSF), radius gyration (Rg), and solvent-accessible surface area (SASA). The RMSD was calculated to examine the overall stability of the docked protein-ligand complex and to monitor the conformational fluctuations during the simulation. The ligand RMSD values for each EGFR-Cryptotanshinone complex were analyzed across the three independent molecular dynamics (MD) simulations (Fig. 8). The complex exhibited substantial fluctuation during the initial 0–10 ns, which likely corresponds to ligand conformational adjustment as the system approached equilibrium. After approximately 20 ns, the RMSD values established and remained consistent until 50 ns, indicating that the ligand achieved a stable binding conformation and maintained a persistent interaction with the protein backbone.

**Fig. 9:**
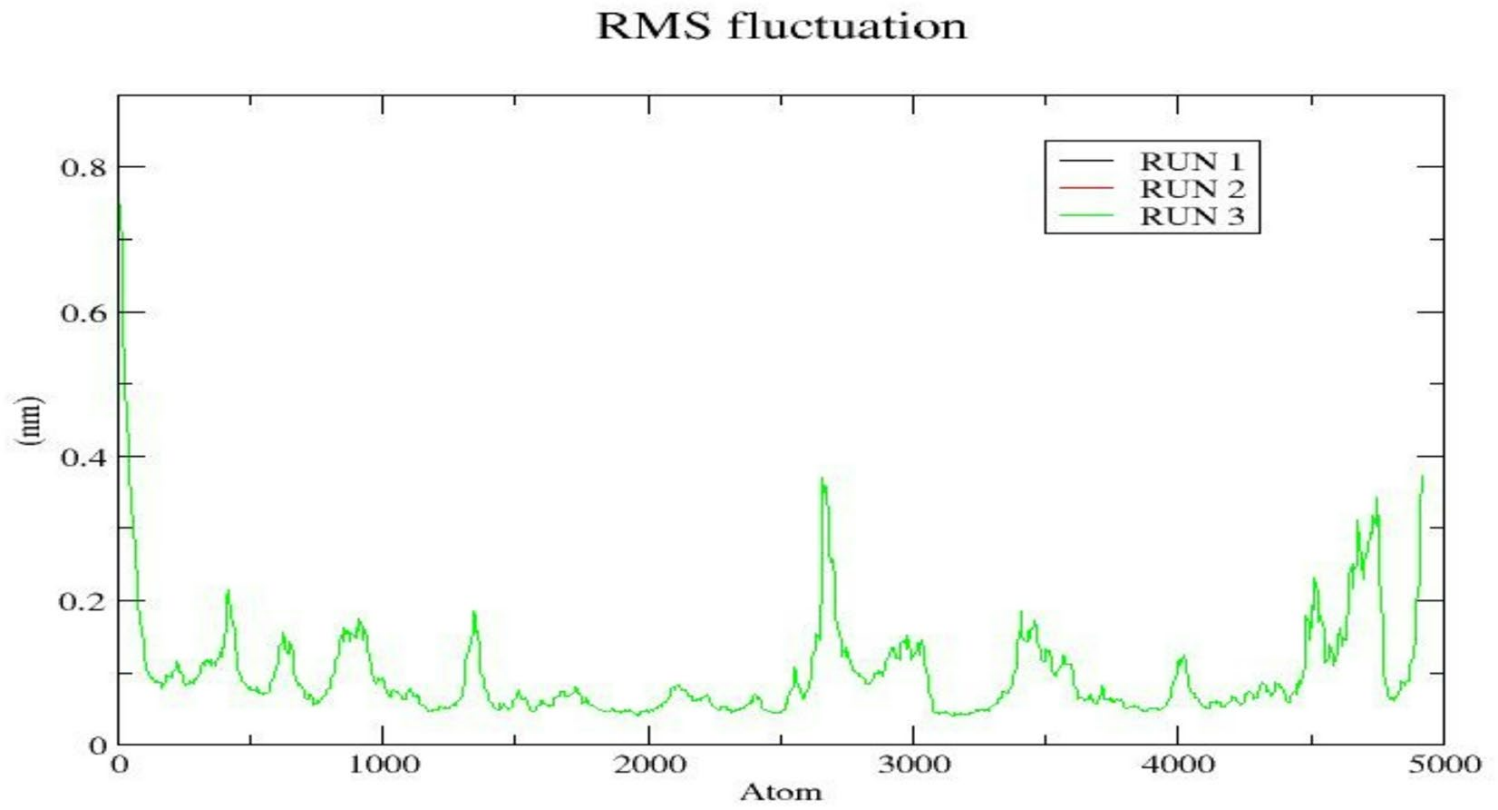
RMSF in residue atoms in the EGFR-Cryptotanshinone complex across triplicate MD simulation runs.

To further examine the flexibility of the EGFR-Cryptotanshinone complex, root mean square fluctuation (RMSF), analysis was conducted to evaluate the residue-wise and backbone dynamics during simulation. The RMSF profiles obtained from three independent MD simulation runs (Fig. 9) revealed that most residues exhibited fluctuations between 0.1 and 0.3 nm, reflecting overall structural stability of the protein-ligand complex. Relatively higher fluctuations ( ⁓0.4–0.8 nm) were observed in loop and terminal regions, indicating localized flexibility in these areas. The consistent RMSF pattern across all replicates demonstrate reproducible dynamics behavior, while the limited fluctuation in the active site residues suggest that Cryptotanshinone binding contributes to maintaining the conformational stability of the EGFR backbone.

**Fig. 10:**
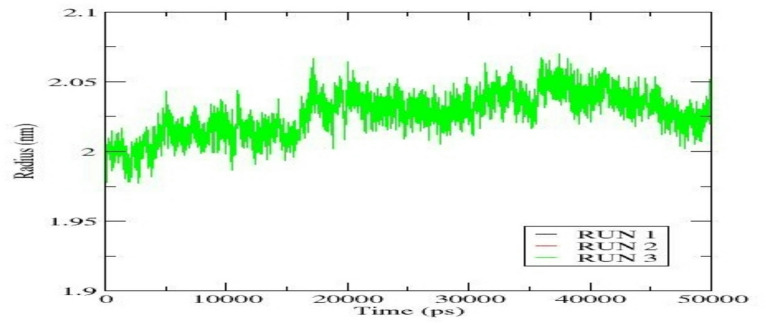
Radius of gyration (Rg) of C-α backbone atoms of the EGFR-Cryptotanshinone complex across triplicate MD simulation runs.

The radius of gyration (Rg) was calculated to evaluate the overall compactness and structural integrity of the EGFR- Cryptotanshinone complex during 50 ns molecular dynamics (MD simulation, performed in triplicate. Through the simulation, the Rg values fluctuate slightly between 1.98 and 2.07 nm, indicating that the overall size and compactness of the EGFR-Cryptotanshinone complex remained stable (Fig. 10). A minor increase in Rg during the first 20 ns suggests initial structural relaxation, associated with the system’s adaptation to the ligand binding. After equilibration, the Rg values stabilized around ⁓ 2.03 nm, signifying that the complex maintained a consistent conformation without significant unfolding. The close overlap among the three runs demonstrated the stability of the EGFR-Cryptotanshinone complex under dynamic conditions.

**Fig. 11:**
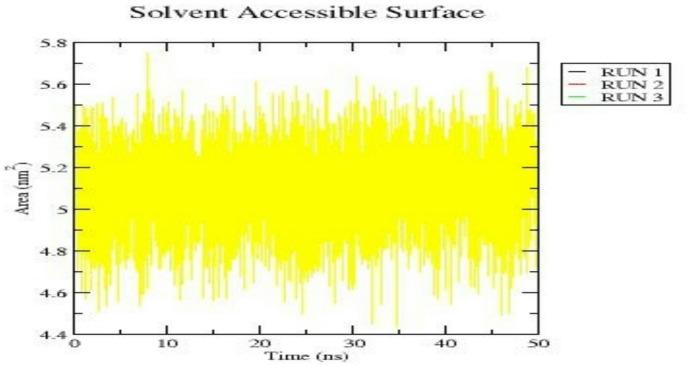
Solvent-accessible surface area (SASA) profile of the EGFR-Cryptotanshinone complex across triplicate MD simulation runs.

The solvent accessible surface area (SASA) analysis was conducted to assess changes in the protein surface exposure upon cryptotanshinone binding to EGFR (Fig. 11). Throughout the 50 ns molecular dynamics simulation, the SASA values fluctuated moderately between 4.6 and 5.6 nm^2^, indicating that the complex maintained a stable solvent-exposed surface. The absence of any significant upward and downward trend suggests that no major conformational expansion occurred during the simulation. The consistent SASA profiles across the triplicate runs further support the structural stability and compactness of the EGFR-cryptotanshinone complex under dynamic conditions.

The accurate binding free energy estimation was calculated from Molecular Mechanics General Born/Poisson-Boltzmann Surface Area (MM-GB/PBSA). This method of binding energy prediction was selected as it balances accuracy and computational power. The gmx_MMPBSA was used to separately minimize the receptor, ligand, and receptor-ligand complex. The EGFFR-Cryptotanshinone complex showed ∆G__bind_ of -34.37 kcal/mol, -29.38 kcal/mol, and − 34.37 kcal/mol for runs 1, 2, and 3.

### In silico cytotoxicity prediction

Table 9 shows the in-silico cytotoxicity prediction and analysis. Compounds were evaluated based on the probability of being active (PA) and inactive (PI) across various cancer cell lines, tumor types, and tissues. Usnic acid exhibited a high probability of cytotoxic activity with 0.767 PA and a low inactivity probability of 0.005 against the U-251 oligodendroglioma cell line located in brain glioma. Anisomycin showed moderate cytotoxic activity with 0.472 PA and 0.068 PI against NCI-H838 small cell lung carcinoma.

**Table 9:** In silico cytotoxicity prediction and analysis of bioactive compounds from *D. meyeniana.*

Code	Bioactive compound	PA	PI	Cell line	Cancer type/CELL type	TISSUE	TUMOR TYPE
A	Usnic acid (CID: 5646)	0.767	0.005	U-251	Oligodendroglioma	Brain	Glioma
B	Anisomycin (CID 253602)	0.472	0.068	NCl-H838	Small-cell lung carcinoma	Lung	Carcinoma
C	5,6,2’-trimethoxyflavone (CID: 14484690)	0.45	0.029	HepG2	Hepatoblastoma	Liver	Hepatoblastoma
D	Cinchonine (CID: 90454)	0.436	0.003	5637	Urothelial bladder carcinoma	Urinary Tract	Carcinoma
E	8,8-dimethyl-2,10-dioxo-9 H-pyrano[2,3-f] chromen-9-yl) (Z)-2-methylbut-2-enoate (CID: 51136479)	0.556	0.021	HL-60	Promyeloblast leukemia	Lymphoid	Leukemia
F	7-[(2E,5E)-7-hydroxy-3,7-dimethylocta-2,5-dienoxy] chromen-2-one (CID: 25763650)	0.36	0.114	NCl − 187	Small-cell lung carcinoma	Lung	Carcinoma
G	4,5-dihydroxy-4,5,6-trimethyl-2,8-dioxa-13-azatricyclo [8.5.1.013,16] hexadec-10-ene-3,7-dione	0.711	0.021	A549	Lung carcinoma	Lung	Lung
H	Cryptotanshinone (CID: 160254)	0.536	0.023	HL-60	Promyeloblast leukemia	Hematopoietic and lymphoid tissue	Leukemia
		0.280	0.007	SW1573	Lung Carcinoma	Lung	Carcinoma
I	6-(1,1-dimethyallyl)-2-(1-hydroxyl-1-methylethyl)-2,3-dihydro-7 H-furo[3,2-G] chromen-7-one (CID: 151467)	0.553	0.021	HL-60	Promyeloblast leukemia	Hematopoietic and lymphoid tissue	Leukemia

PA: probability of being active; PI: probability of being inactive.

## Discussion

*D. meyeniana* possesses a wide range of pharmacological properties due to its diverse chemical secondary metabolites, as revealed through GC–MS and UHPLC-QTOF-MS analyses. The identified compounds include sesquiterpenoids, terpenoids, fatty acids, and Silphinene, and each associated with significant biological activities. Sesquiterpenoids are well-documented for their biological activities, exhibiting antitumor, antibacterial, anti-inflammatory, anti-viral^61^, and anticancer properties^62^. Fatty acids, characterized by a long hydrocarbon chain and a terminal carboxyl group, have demonstrated inhibitory effects on carcinogenesis in various tumor cell lines^63^.

Similarly, terpenoids are recognized for their ability to interfere with multiple stages of cancer progression, contributing to their anticancer properties. Among the volatile constituents, Stigmast-5-ene,3β-(trimethlysiloxy)-,24s is the most abundant compound. This molecule has been reported to exert strong anticancer activity by inducing apoptosis, causing cell arrest, inhibiting angiogenesis, and suppressing metastasis. Its mechanism involves targeting specific proteins such as Na+/K + ATPase, leading to disruption of cancer survival pathways^64^. The second most prominent compound was Phytol, a diterpenoid, which is known for its pro-apoptotic and necrotic effects on sarcoma 180 and human leukemia carcinoma cells^65^.

The detected fatty acids, such as palmitic acid, α-linolenic acid, and linoleic acid, are linked to apoptosis induction, inhibition of cell migration, invasion, and angiogenesis^66–68^. Linoleic acid further supports cellular metabolism and holds therapeutic potential. Other fatty acids, including Docosanol, 6-hydroxypalmitic acid, and Coriolic acid, also exhibit anticancer properties. Docosanol inhibits CHO-K1 and melanoma cell lines^69^, 6-hydroxypalmitic acid is a candidate for anticancer drug development^70^, and Coriolic acid targets breast cancer stem cells by downregulating c-Myc^64^.

Additional compounds like Epoxy-octadecenoic acid, Decylbenzenesulfonic acid, and Hydroxy-octadecadienoic acid may also contribute to anticancer effects. Flavonoids, known for their antiproliferative, pro-apoptotic, and antiangiogenic properties^71^, include 5,6,2’-trimethoxyflavone, which shows activity against Aspc-1 pancreatic cancer cells^72^. Another compound, methyl 2-[(3,4-diethoxyphenyl)methylene]-3-oxobenzo[b]furan-5-carboxylate, adds to the anticancer potential. Terpenoids, bioactive molecules with mechanisms such as mitochondrial apoptosis induction and PI3K/AKT and NF-κB pathway inhibition^73^, include Uracaric acid and Cryptotanshinone. Cryptotanshinone notably inhibits lung cancer invasion, tumorigenesis, and proliferation^74^. Coumarins also exhibit strong anticancer activity by regulating the cell cycle, angiogenesis, apoptosis, and autophagy^75^. Key coumarins identified include derivatives such as 8-8-dimethyl-2,10-dioxochromen and 6-(1,1-dimethylallyl)-2,3-dihydrochromen. The diverse phytochemicals in *D. meyeniana* highlight its potential as a promising source for anticancer drug development. Silphinene has demonstrated strong binding affinity to cancer-related proteins, including ERG, HER2, ABL1, and P13K-α, suggesting its potential as a molecular inhibitor in cancer therapy^76^. Neophytadiene, another bioactive compound, exhibited a broad pharmacological spectrum, including anti-inflammatory, antioxidant^77^, antimicrobial, and anticancer^78^. Collectively, these findings highlight that D. *meyeniana* is a rich source of bioactive compounds with significant anticancer potential.

Non-volatile compounds profiling showed alkaloids, fatty acids, flavonoids, terpenoids, and coumarins, each associated with significant anticancer properties. Alkaloids such as Anisomycin, Cinchonine, Indole-3- caboxaldehyde, 3-methyl-8,10,20,22-tetraoxa-3-azapentacyclo[15.7.0.05,13.07,11.019.23]tetracosa-1(17),5,7(11),12,18,23-hexaen-14-one and monocrotaline were identified in the extract. Alkaloids are widely recognized for their antiproliferative and cytotoxic effects, with several already established as chemotherapeutic agents, including vinblastine, vinorelbine, vincristine, and vindesine^79^. Specifically, Anisomycin inhibits the growth, survival, and migration of non-small cell lung cancer^80^ while Cinchonine disrupts autophagy by blocking autophagosome degradation via the inhibition of lysosomal hydrolases maturation^81^. Indole-3-carboxaldehyde has also demonstrated both antimycobacterial and anticancer agents^82^.

In the early stages of drug development, assessing physicochemical properties, pharmacokinetics, drug-likeness, and medicinal chemistry characteristics is essential for identifying promising therapeutic candidates. This evaluation optimizes a compound’s potential toxicity and helps prevent time-consuming and costly failures in later clinical trials. For a bioactive compound to qualify as a potential drug candidate, it must not only demonstrate the desired biological activity but also possess favorable pharmacokinetic properties^74^. In this study, we utilized in silico approaches to evaluate the pharmacokinetics, drug-likeness, and key medicinal chemistry properties of bioactive compounds derived from *D. meyeniana*.

Lipophilicity, which indicates a compound’s ability to dissolve in lipids and nonpolar solvents, is crucial for determining its ADMET profile. It impacts distribution, metabolism, elimination, and membrane interactions. An optimal lipophilicity range of 0 to 5 is recommended for drug development^83,84^. In this analysis, all nine compounds exhibited favorable lipophilicity, suggesting efficient membrane permeability and systemic bioavailability.

Solubility also plays a significant role in drug absorption and distribution^84^. Effective drug absorption requires sufficient solubility in aqueous solutions at the site of uptake^74^. Eight compounds showed moderate solubility, indicating good oral bioavailability potential, while Anisomycin demonstrated high solubility, which may necessitate formulation strategies for optimal systemic circulation.

ADME properties are essential for understanding a compound’s pharmacokinetic behavior and influence on pharmacological activity. Efficient gastrointestinal absorption is critical for orally administered drugs. This study revealed that all nine compounds had good absorption potential via the gastrointestinal tract, with most predicted to cross the blood-brain barrier (BBB). However, Usnic acid and 8-8-dimethyl-2,10-dioxo-9 H-pyrano2,3-fchromen-9-yl (Z)-2-methylbut-2-enoate were less likely to penetrate the BBB, potentially reducing neurotoxic risks.

P-glycoprotein (P-gp) functions as an ATP-dependent efflux transporter that limits intracellular drug accumulation by actively transporting xenobiotics and therapeutic agents out of cells, thereby reducing their plasma and tissue concentrations. In the present study, seven of the tested compounds were predicted to be non-substrates of P-gp, except cinchonine and cryptotanshinone, which may be subject to P-gp-mediated efflux, potentially lowering their intercellular availability and therapeutic efficacy. Cytochrome P450 (CYP) isoenzymes also play a pivotal role in determining pharmacokinetic behavior, as they are primarily responsible for the metabolism and clearance of xenobiotics. Inhibition of CYP isoforms can impede drug metabolism, potentially resulting in elevated plasma concentration and toxicity. The current analysis revealed that usnic acid, anisomycin, and the reference drug erlotinib exhibited strong inhibitory potential against major CYP isoforms, whereas eprenetapopt (APR-246) showed no inhibitory potential against major CYP enzymes. The latter finding suggests that eprenetapopt is likely to undergo effective hepatic metabolism and clearance, thereby minimizing the risk of toxicity and adverse pharmacokinetic interactions^84^.

Drug-likeness evaluates a compound’s potential for clinical development by assessing the alignment of its physicochemical and structural features with those of approved drugs. This serves as a valuable tool for reducing research costs by eliminating compounds with poor pharmacokinetic profiles^85^. In this study, five well-known computational filters - Lipinski, Ghose, Veber, Egan, and Muegge - were used to assess drug-likeness, revealing that all nine compounds met the criteria, indicating promising characteristics for further development.

The compounds were also screened for pan-assay interference compounds (PAINS) and BRENK alerts. Except for cryptotanshinone, none triggered PAINS alerts, suggesting the absence of structural motifs that could cause false positives during in silico screening^74^. However, several compounds displayed BRENK alerts, indicating potentially toxic structural fragments, particularly Usnic acid and Anisomycin. While such alerts require attention, they do not disqualify compounds and can often be addressed through structural optimization in lead development.

Pro-Tox-II was used to assess the cytotoxicity of selected compounds from *D. meyeniana*, examining acute toxicity (LD50 values), organ toxicity, genotoxicity, and carcinogenicity. The compounds 5,6,2’-trimethoxyflavone, 7-(2E,5E)-7-hydroxy-3,7-dimethylocta-2,5-dienoxychromen-2-one, 5-dihydroxy-4,5,6-trimethyl-2,8-dioxa-13-azatricyclo [8.5.1.013,16] hexadec-10-ene-3,7-dione, and Cryptotanshinone all exhibited LD50 values higher than 2000 mg/kg and were not active in organ toxicity, except for Cryptotanshinone, which showed carcinogenic activity.

The bioavailability radar plot visually represents drug-like characteristics by evaluating six key pharmacokinetic properties: lipophilicity, size, polarity, solubility, flexibility, and saturation^2^. The pink area indicates optimal drug-likeness, defining acceptable ranges for key parameters. Eight bioactive compounds from this study - Usnic acid, Anisomycin, Cinchonine, 8,8-dimethyl-2,10-dioxo-9 H-pyrano2,3-fchromen-9-yl(Z)-2-methylbut-2-enoate, 7-(2E,5E)-7-hydroxy-3,7-dimethylocta-2,5-dienoxychromen-2-one, 4,5-dihydroxy-4,5,6-trimethyl-2,8-dioxa-13-azatricyclo [8.5.1.013,16] hexadec-10-ene-3,7-dione, Cryptotanshinone, and 6-(1,1-dimethyallyl)-2-(1-hydroxyl-1-methylethyl)-2,3-dihydro-7 H-furo3,2-Gchromen-7-one - fell entirely within this optimal region of the of the bioavailability radar, indicating strong potential for favorable oral bioavailability. In contrast, 5,6,2’-trimethoxyflavone was positioned outside the acceptable range for saturation, suggesting potential limitations in oral bioavailability profile compared with the standard anticancer agent Eprenetapopt (APR-246), and was comparable to Erlotinib, which partially falls outside the optimal zone^86^. This suggests that these phytochemicals are likely capable of achieving efficient systemic absorption and distribution, supporting their potential as promising drug candidates^87^.

Among the evaluated compounds, Cryptotanshinone demonstrated the most favorable ADMET characteristics and drug-likeness profile, justifying further investigation of its anticancer potential through molecular docking. This established in silico modeling technique assesses the interaction between small-molecule ligands and proteins^88^, with binding energy serving as a measure of affinity, in which lower energies indicate stronger interactions. Docking analysis focused on four key cancer-related proteins: epidermal growth factor receptor (EGFR), tumor suppressor protein p53, matrix metalloproteinase-7 (MMP7), and cyclin-dependent kinase 8/Cyclin C (CDK8/Cyclin C). EGFR, a transmembrane receptor tyrosine kinase, mediates signals in cell proliferation, differentiation, and migration^89^. The p53 protein prevents phenotypic and genomic changes linked to cancer progression^90^. MP7 facilitates cancer cell invasion and angiogenesis via extracellular matrix degradation^91^, while CDK8 regulates transcription and cell cycle progression, influencing tumor development^92^. These targets were chosen due to their significant roles in oncogenesis and cancer progression. The strong binding affinity of Cryptotanshinone for these proteins emphasizes its potential as a multi-target anticancer agent.

Cryptotanshinone exhibited strong binding affinity toward all four target proteins, forming multiple stabilizing interactions within or near their respective active sites. In the EGFR complex, cryptotanshinone engaged in ℼ-sigma and alkyl interaction that facilitated intercalation within the binding pocket, contributing substantially to complex stability. Its interaction with tumor suppressor protein p53 was reinforced by alkyl chain contacts and ℼ-cation interaction, further strengthening ligand binding. In the MMP7 complex, the formation of conventional hydrogen bonds enhanced the structural stability of the ligand–protein complex. Although Cryptotanshinone did not directly interact with the catalytic residues of cyclin-dependent kinase 8/ Cyclin C (CDK8/Cyclin C) it established hydrogen bonds and van der Waals contacts with residues adjacent to the active site cleft^74^. These interactions may induce local conformational changes that interfere with enzymatic activity. Overall, the docking results revealed that van der Waals forces were the primary contributors to the complex stabilization, supported by hydrophobic interactions, π-alkyl, and π-π stacking interactions^93^. Collectively, these non-covalent interactions enhance binding affinity and selectivity, suggesting that Cryptotanshinone may serve as a potent inhibitor of these cancer-associated proteins. Cryptotanshinone consistently emerged as the most promising compound, exhibiting robust binding interactions across all targets. These findings highlight the therapeutic potential of *D. meyeniana*-derived phytochemicals as novel anticancer agents, possibly with reduced adverse effects compared to the conventional chemotherapy drugs, Erlotinib and Eprenetapopt (APR-246). However, further experimental validation and structural optimization are essential to confirm efficacy and optimize pharmacological profiles for future drug development^94,95^. CLC-Pred, based on the Prediction of Activity Spectra for Substances (PASS) algorithm, was employed to estimate the biological activity profile of cryptotanshinone. The PASS tool utilizes a leave-one-out cross-validation approach, with a reported predictive accuracy of approximately 96% compared to experimental in vivo data. The analysis predicted a notable cytotoxic potential of cryptotanshinone against promyeloblast leukemia cells, further supporting its relevance as a candidate for anticancer drug development. Overall, cryptotanshinone consistently emerged as the most promising compound, exhibiting robust binding interactions across all evaluated cancer-related targets^94,95^. Advancements in computational techniques and structure-based drug design have become essential for predicting the pharmacokinetic properties and drug-like profiles of natural products^96^.

Molecular dynamics (MD) simulations were conducted to further validate the stability of Cryptotanshinone in complex with cancer-related proteins, with the epidermal growth factor receptor (EGFR) selected as a representative target^97^. The stability analyses included root mean square deviation (RMSD), root mean square fluctuation (RMSF), solvent-accessible surface area (SASA), and radius of gyration (Rg) calculations. Moderate RMSD values throughout the simulation indicate minimal conformational deviations, suggesting that the protein-ligand complex remained stable over the simulation period. THE RSMF profile revealed limited fluctuations at key binding residues, signifying strong and consistent intermolecular interactions. Similarly, SASA analysis demonstrated that Cryptotanshinone maintained a compact binding conformation, minimizing solvent exposure and reinforcing the stability of the binding pocket. The Rg analysis further supported these findings, exhibiting minimal variation and confirming the structural compactness of the complex^37^. The triplicate simulation runs consistently showed stable complex formation, verifying the reproducibility and robustness of the observed interactions. The MM-GBSA calculations revealed favorable binding free energies, primarily driven by van der Waals interactions, indicating that hydrophobic forces significantly contribute to the stability of the Cryptotanshinone-EGFR complex^36^. Collectively, these results highlight the strong binding potential and structural stability of Cryptotanshinone against cancer-associated targets. As a naturally derived compound, Cryptotanshinone offers additional advantages such as cost-effectiveness, sustainability, and environmental friendliness, further supporting its potential as a therapeutic lead.

Despite these promising findings, several limitations must be acknowledged. This study is primarily based on computational approaches, including molecular docking, ADMET prediction, and molecular dynamics simulation, which, while powerful for early-stage screening, cannot fully capture the complexity of the in vivo biological system. Factors such as metabolism, enzymatic interactions, immune responses, and interindividual differences were not addressed in the present analysis. Moreover, although Cryptotanshinone demonstrated a low predicted cytotoxicity in lung cancer models, these predictions are driven solely by in silico data. Therefore, experimental validation through in vitro and in vivo assays is essential to confirm the binding affinities, pharmacokinetic behavior, anticancer efficacy, and potential off-target effects of this compound.

Cryptotanshinone has also been identified as a P-glycoprotein (P-gp) substrate, which may limit its oral bioavailability due to efflux-mediated transport. Future studies should explore formulation or chemical modification strategies to overcome this limitation. Additionally, while four cancer-related proteins were screened, EGFR was chosen for detailed simulation because of its pivotal role in the progression of multiple cancer types. Expanding the investigation to other signaling pathways could provide a broader understanding of the therapeutic scope and safety profile of Cryptotanshinone. Erlotinib and Eprenetapopt (APR-246) were used as reference compounds in this study; direct experimental comparison with Cryptotanshinone was not performed. This limitation restricts the ability to assess the clinical superiority or complementary potential of Cryptotanshinone relative to existing drugs. Furthermore, critical factors relevant to clinical development, such as chemical stability, scalability of production, and formulation challenges, were not addressed. These aspects warrant further investigation to facilitate the translation of Cryptotanshinone into viable therapeutic applications.

## Conclusion

*Dendrocnide meyeniana* exhibits notable phytochemical diversity, emphasizing its potential as a source of bioactive compounds for anticancer research. Among its identified metabolites, Cryptotanshinone demonstrated strong binding affinity and structural stability toward multiple cancer-associated protein targets, showing binding efficacy comparable to the clinically approved EGFR inhibitor Erlotinib.

Molecular dynamics simulation further validated the stability of the Cryptotanshinone-EGFR complex, revealing consistent conformational integrity supported by favorable van der Waals, π-alkyl, and π–π interactions. Stable RMSD, compact Rg, and limited RMSF fluctuationss confirmed durable binding within the target pocket under near-physiological conditions.

The convergence of docking and MD results supports a reliable binding mechanism, positioning Cryptotanshinone as a promising lead compound for plant-derived anticancer drug development. Nevertheless, as this study is based on computational predictions, comprehensive experimental validation - including in vitro, in vivo, and pharmacokinetic assessments - is essential to confirm its efficacy, safety, and therapeutic potential.

## Supplementary Information

Below is the link to the electronic supplementary material.

Supplementary Material 1

## Data Availability

The data presented in this study are available in this article. Further inquiries can be directed to the corresponding authors.
