# Supplementary material for: Anticancer potential of Dendrocnide meyeniana through phytochemical profiling, ADMET analysis, molecular docking, and in silico cytotoxicity evaluation
Source: Sci Rep. 2025 Dec 27;16:2704. doi: 10.1038/s41598-025-32457-1 (PMC12823615; doi:10.1038/s41598-025-32457-1)
Supplement: Supplementary file 1 — Supplementary Material 1 [file 41598_2025_32457_MOESM1_ESM.pdf]

Supplementary Table 1. The GC-MS analysis results of *D. meyeniana.*

Peak	RT	MW	Tentative compound	% Peak area	MF	Nature of Compound
1	7.021	90.08	Lactic acid*	0.29	$C_3H_6O_3$	Organic compound
2	8.206	60.056	Urea*	0.57	CH_4N_2O	Organic acid
3	9.671	106.12	Diethylene glycol*	0.53	$C_4H_{10}O_3$	Organic compound
4	10.157	92.09	Glycerol*	3.74	$C_3H_8O_3$	Carbohydrate
5	10.884	204.35	Silphiperfol-5-ene	0.39	$C_{15}H_{24}$	Sesquiterpenoid
6	11.176	204.35	Silphinene	3.75	C_1H_{245}	Sesquiterpenoid
7	11.668	204.35	Modephene	0.62	$C_{15}H_{24}$	Sesquiterpenoid
8	11.754	204.35	alpha-isocomene	0.41	$C_{15}H_{24}$	Sesquiterpenoid
9	12.04	204.35	(±)-β-Isocomene	0.40	$C_{15}H_{24}$	Sesquiterpenoid
10	12.171	204.35	Caryophyllene	0.40	$C_{15}H_{24}$	Sesquiterpenoid
11	13.298	122.12	Meso-erythritol	1.90	$C_4H_{10}O_4$	Carbohydrate
			L-Threitol*		$C_4H_{10}O_4$	Carbohydrate
12	13.625	206.32	2,4-Di-tert-butylphenoxytrimethylsilane	0.51	$C_{14}H_{22}O$	Phenylpropane
13	15.828	152.15	D- (+)-arabitol	1.83	$C_5H_{12}O_5$	Carbohydrate
			L- (-)-arabitol		$C_5H_{12}O_5$	Carbohydrate
			Xylitol*		$C_5H_{12}O_5$	Carbohydrate
14	15.885	152.15	Adonitol*	0.48	$C_5H_{12}O_5$	Carbohydrate
15	16.812	278.5	Neophytadiene	2.96	$C_{20}H_{38}$	Sesquiterpenoid
16	16.915	228.37	Myristic acid*	1.13	$C_{14}H_{28}O_2$	Fatty acid
17	17.064	280.5	2-hexadecen-1-ol, 3,7,11,15tetramethyl-, acetate, [R- [R*, R*-(E)]]-	1.21	$C_{20}H_{40}$	Fatty acid
18	17.252	296.53	3,7,11,15-tetramethyl-2-hexadecen-1ol	1.69	$C_{20}H_{40}O$	Isoprenoid
19	17.481	180.16	D-fructose, 1,3,4,5,6-pentakis-o(trimethylsilyl)-, o-methyl oxime	1.41	$C_{16}H_{12}O_6$	Organooxygen compound
20	17.584	180.16	D- (-)-fructose, pentakis(trimethylsilyl) ether, methyloxime (syn)	0.34	$C_{16}H_{12}O_6$	Organooxygen compound
21	17.745	180.16	D-glucose, 2,3,4,5,6-pentakis-O-(trimethylsilyl)-, o-methyloxyme, (1Z)-	0.85	$C_{16}H_{12}O_6$	Organooxygen compound
22	18.054	291.435	2,4,6 tri-tert-butylnitrobenzene	3.74	$C_{18}H_{36}O_2$	Aromatic nitro compound
23	18.340	284.477	Hexadecenoic acid, ethyl ester	1.11	$C_{18}H_{36}O_2$	Fatty acid
24	18.855	256.42	Palmitic acid	10.28	$C_{16}H_{36}O_2$	Fatty acid
25	20.068	296.50	Phytol	11.73	$C_{20}H_{40}O$	Diterpene
26	20.382	280.45	9,12- octadecadienoic acid	4.87	$C_{18}H_{32}O_2$	Fatty acid
27	20.455	278.40	ά-linolenic acid	3.81	$C_{18}H_{32}O_2$	Fatty acid
28	20.628	284.50	Stearic acid	2.14	$C_{18}H_{32}O_2$	Fatty acid
29	22.271	312.50	Arachidic acid	0.64	$C_{20}H_{40}O_2$	Fatty acid
30	24.250	342.30	Sucrose	0.37	$C_{12}H_{22}O_{11}$	Carbohydrate
31	25.017	342.30	Lactose	0.47	$C_{12}H_{22}O_{11}$	Carbohydrate
32	25.767	402.70	δ-tocopherol	1.94	$C_{27}H_{46}O_2$	Quinone and
33	26.522	416.70	γ-tocopherol	0.36	$C_{28}H_{48}O_2$	Quinone and
34	29.183	400.70	Campesterol	1.55	$C_{28}H_{48}O$	Ergosterol
35	30.350	484.87	Stigmast-5-ene,3β-(trimethlysiloxy)-,24s	26.99	$C_{32}H_{56}O$	Triterpenes
36	30.533	412.70	Isofucosterol	2.36	$C_{29}H_{48}O$	Sterol lipid
37	31.231	426.71	9,19-Cyclolanost-24-en-3-ol,(3β)-,	0.99	$C_{30}H_{50}O$	Triterpenoid
38	32.147	440.74	9,19-Cyclolanost-24-en-3-ol, 24-methylene-(3β)-,	0.41	$C_{31}H_{52}O$	Triterpenoid
39	32.885	426.72	Citrostadienol*	0.42	$C_{30}H_{50}O$	Triterpenoid

*Tentatively identified compound was derivatized with methoxyamine HCl in pyridine and BSTFA with 1% TMCS.

Supplementary Table 2. Phytochemical Profile of *D. meyeniana* using UHPLC-QTOF-MS.

ID	RT (min)	m/z	Tentative compound	MW (g/mol)	MF	Cosine Similarity	Nature of Compound
1	23.29	325.30	Docosanol	326.6	$C_{22}H_{46}O$	0.96	Fatty acid
2	19.61	343.08	Usnic acid	344.3	$C_{18}H_{16}O_7$	0.96	Polyketides
3	21.65	297.14	Decylbenzenesulfonic acid	298.4	$C_{16}H_{26}O_3S$	0.96	Polyketides
4	16.61	264.10	Anisomycin	265.3	$C_{14}H_{19}NO_4$	0.96	Alkaloid
5	19.95	311.10	5,6,2'-trimethoxyflavone	312.3	$C_{18}H_{16}O_5$	0.95	Flavonoid
6	20.24	293.16	Cinchonine	294.4	$C_{19}H_{22}N_2O$	0.94	Alkaloid
7	23.95	271.23	16-hydroxypalmitic acid	272.42	$C_{16}H_{32}O_3$	0.94	Fatty acid
8	20.25	293.20	13S-hydroxy-6Z,9Z,11E-octadecatrienoic acid13(S)	294.4	$C_{18}H_{30}O_3$	0.93	Fatty acid
9	27.35	341.10	8,8-dimethyl-2,10-dioxo-9h-pyrano[2,3-f] chromen-9-yl) (Z)-2-methylbut-2-enoate	342.3	$C_{19}H_{18}O_6$	0.93	Coumarin
10	23.05	483.30	1-hexadecanoyl-2-(9z-octadecenoyl)-sn-glycero-3-phospho-(1'-rac-glycerol)	749	$C_{40}H_{77}O_{10}P$	0.92	glycerophosphol
11	21.13	311.20	Benzenesulfonic acid, 4-undecyl-	312.5	$C_{17}H_{28}O_3S$	0.92	Benzenesulfonic
12	10.14	144.00	Indole-3- carboxaldehyde	145.2	C_9H_7NO	0.89	Alkaloid
13	14.88	487.343	(1R,2R,4aS,6aS,6bR,10S,12aR,14bS)-1,8,10-trihydroxy-1,2,6a,6b,9,9,12a-	488.7	$C_{30}H_{48}O_5$	0.85	Triterpenoid
14	21.65	295.20	9,10-eode|9,10-epoxy-12z-octadecenoic acid| (+-)9(10)-	296.4	$C_{18}H_{32}O_3$	0.83	Fatty acid
15	14.80	487.30	(1R,2R,4aS,6aS,6bR,9R,10R,11R,12aR)-1,10,11-trihydroxy-9-(hydroxymethyl)-1,2,6a,6b,9,12a-hexamethyl-2,3,4,5,6,6a,7,8,8a,10,11,12,13,14b-tetradecahydropicene-4a-carboxylic acid. (Uncaric acid)	504.7	$C_{30}H_{48}O_6$	0.83	Triterpenoid
16	21.65	297.14	Decylbenzenesulfonic acid	298.4	$C_{16}H_{26}O_3S$	0.81	Fatty acid
17	21.88	295.20	9-hydroxy-10,12-octadecadienoic acid	296.4	$C_{18}H_{32}O_3$	0.81	Fatty acid
18	8.29	352.1	3-methyl-8,10,20,22-tetraoxa-3-azapentacyclo [15.7.0.05,13.07,11.019,23] tetracosa-1(17),5,7(11),12,18,23-hexaen-14-one	No available	$C_{20}H_{19}NO_5$	0.81	Alkaloids
19	17.57	535.20	(1R,2R,4S,7S,8R,9R,10S,11R,12S,13S,14R,17R,18R,19R)-8-Acetoxy-10,19-dihydroxy-1,9,18-trimethyl-15-oxo-16,20-dioxahexacyclo [15.3.2.0~2,13~.0~4,12~.0~7,11~.0~14,19~] docos-5-ene-5-carboxylic acid	490.5	$C_{26}H_{34}O_9$	0.80	Terpenoid
21	18.2	311.1	5,6,2'-trimethoxyflavone	312.3	$C_{18}H_{16}O_5$	0.79	Flavonoid
22	21.65	368.10	methyl 2-[(3,4-diethoxyphenyl) methylene]-3-oxobenzo[b]furan-5-carboxylate	368.4	$C_{21}H_{20}O_6$	0.78	Flavonoid
23	19.61	138.01	4-nitrophenol	139.11	$C_6H_5NO_3$	0.76	Phenolic
24	30.68	324.10	4,5-dihydroxy-4,5,6-trimethyl-2,8-dioxa-13-azatricyclo [8.5.1.013,16] hexadec-10-ene-3,7-dione	314.4	$C_{19}H_{22}O_4$	0.77	Ornithine
25	29.03	134.90	7-[(2E,5E)-7-hydroxy-3,7-dimethylocta-2,5-dienoxy]chromen-2-one	314.4	$C_{19}H_{22}O_4$	0.76	Coumarin
26	7.42	297.14	Cryptotanshinone	296.4	$C_{19}H_{20}O_3$	0.75	Diterpenoid
27	21.38	313.50	6-(1,1- dimethylallyl)-21-hydroxy-1-methylethyl)-2,3-dihydro-7H-furo[3,2-G] chromen-7-one	314.4	$C_{19}H_{22}O_4$	0.75	Coumarin
28	11.57	372.94	6-O-methylarthothelin	375.6	$C_{15}H_9C_{13}O_5$	0.74	Xanthone
29	16.14	329.23	(10E,12E,14E)-16-hydroxy-9-oxooctadeca-10,12,14-trienoic acid	330.5	$C_{18}H_{34}O_5$	0.74	Octadecanoid
30	9.90	539.10	Cynarin	516.4	$C_{25}H_{24}O_{12}$	0.73	Phenylpropanoi
31	21.88	482.2	2-[hydroxy-[(2R)-2-hydroxy-3pentadecanoyloxypropoxy] phosphoryl] oxyethyl-trimethylazaniu	482.6	$C_{23}H_{49}NO_7P+$	0.72	Phospholipid
32	17.16	710.4	[3-[2-[3-[[10,13-dimethyl-17-(6-methylheptan-2-yl)2,3,4,7,8,9,11,12,14,15,16,17-dodecahydro-	710.9	$C_{39}H_{69}NO_8P-$	0.72	Steroid
33	15.61	539.1	4',4''',5,5",7,7"-hexahydroxy-3,8"-biflavone	538.5	$C_{30}H_{18}O_{10}$	0.72	Flavonoid
34	16.89	307.1	(10E,12E,14E)-16-hydroxy-9-oxooctadeca-10,12,14-trienoic acid	308.4	$C_{18}H_{28}O_4$	0.72	Fatty acid
35	15.6	309.	(E)-4-[(1R,2S,3S,4R,8aS)-2,3,4-trihydroxy-2,5,5,8a-tetramethyl-3,4,4a,6,7,8-hexahydro-1H-naphthalen-1-yl]but-3-en-2-one	310.4	$C_{18}H_{30}O_4$	0.71	Terpenoid
36	18.7	313.2	12,13-Dihome	314.5	$C_{18}H_{34}O_4$	0.71	Fatty acid
37	9.00	356.3	(2S)-2-amino-3-methylbutanoic acid; hexadecenoic acid	373.6	$C_{21}H_{43}NO_4$	0.70	Octadecanoid
39	10.5	343.2	(. +/-.)-4-Hydroxy-5E,7Z,10Z,13Z,16Z,19Z-docosahexaenoic acid	344.5	$C_{22}H_{32}O_3$	0.70	Fatty acid
